# Multi-Omic Analyses of Growth Cones at Different Developmental Stages Provides Insight into Pathways in Adult Neuroregeneration

**DOI:** 10.1016/j.isci.2020.100836

**Published:** 2020-01-14

**Authors:** Muhammad Zain Chauhan, Jennifer Arcuri, Kevin K. Park, Maroof Khan Zafar, Rabeet Fatmi, Abigail S. Hackam, Yuqin Yin, Larry Benowitz, Jeffrey L. Goldberg, Mohammad Samarah, Sanjoy K. Bhattacharya

**Affiliations:** 1 Bascom Palmer Eye Institute, University of Miami Miller School of Medicine, Miami, FL 33136, USA; 2 Miami Integrative Metabolomics Research Center, University of Miami Miller School of Medicine, Miami, FL 33136, USA; 3 Program in Biomedical Sciences & Neuroscience Graduate Program, University of Miami Miller School of Medicine, Miami, FL 33136, USA; 4 Miami Project to Cure Paralysis, University of Miami Miller School of Medicine, Miami, FL 33136, USA; 5 Department of Biochemistry and Molecular Biology, University of Arkansas for Medical Sciences, Little Rock, AR 72205, USA; 6 Department of Computer Science, Florida Polytechnic University, Lakeland, FL 33805, USA; 7 Department of Neurosurgery, Harvard Medical School, Boston, MA 02115, USA; 8 Department of Ophthalmology, Harvard Medical School, Boston, MA 02115, USA; 9 Department of Neurosurgery and F.M. Kirby Neurobiology Center, Boston Children's Hospital, Boston, MA 02115, USA; 10 Department of Ophthalmology, Stanford University School of Medicine, Stanford, CA 94305, USA

**Keywords:** Biological Sciences, Systems Biology, Omics, Proteomics, Lipidomics

## Abstract

Growth cones (GCs) are structures associated with growing neurons. GC membrane expansion, which necessitates protein-lipid interactions, is critical to axonal elongation in development and in adult neuritogenesis. We present a multi-omic analysis that integrates proteomics and lipidomics data for the identification of GC pathways, cell phenotypes, and lipid-protein interactions, with an analytic platform to facilitate the visualization of these data. We combine lipidomic data from GC and adult axonal regeneration following optic nerve crush. Our results reveal significant molecular variability in GCs across developmental ages that aligns with the upregulation and downregulation of lipid metabolic processes and correlates with distinct changes in the lipid composition of GC plasmalemma. We find that these processes also define the transition into a growth-permissive state in the adult central nervous system. The insight derived from these analyses will aid in promoting adult regeneration and functional innervation in devastating neurodegenerative diseases.

## Highlights

• Simultaneous proteomics and lipidomics analyses of developmental growth cones
• Combined multi-omics analyses of regenerating optic nerves and growth cones
• Integrating protein-protein with protein-lipid interactions in growth cones

## Graphical Abstract

**Figure:**
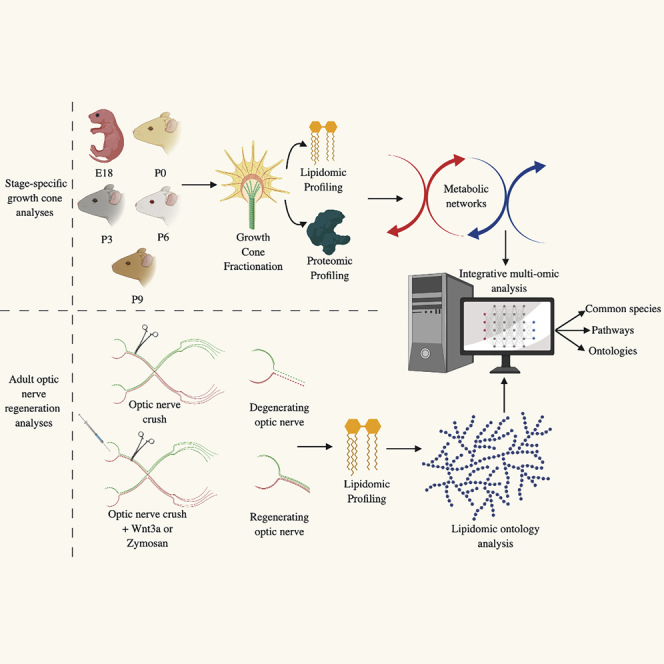

## Introduction

Growth cones (GCs) are terminally enlarged amoeboid-like structures of growing neurons. They are one of a growing neuron's most essential structures, responsible for neuronal expansion toward a target during early development, collateral sprouting resulting in additional or new neuronal connectivity, and enabling regeneration of severed neurites in the central nervous system (CNS) and peripheral nervous system (PNS) in adults. The latter is the fulcrum in devising novel intervention strategies for functional recovery in several progressive neurodegenerative diseases, such as Parkinson disease (Diaz-Martinez et al., 2013), spinal cord injuries, and progressive neuropathies, like glaucoma. The field of neuroscience has made huge leaps from theories about their existence (Cajal, 1890, Garcia-Marin et al., 2009) to their morphological and molecular composition (Tamariz and Varela-Echavarria, 2015), with recent high-throughput studies having focused on proteomics analysis (Estrada-Bernal et al., 2012, Nozumi et al., 2009). However, notably lacking in the GC literature are multi-omic studies at different developmental time points, with no work to date having analyzed the GC lipidome. The expansion of the plasma membrane (or plasmalemma), whose function is a property of lipid and protein interactions (Koberlin et al., 2015), is essential for GC movement and neuroregeneration. Specific lipid species are spread across the plasmalemma in a region-specific manner and provide a distinct functional organization to the GC outer membrane (Holthuis and Menon, 2014). The propagation of GCs, through plasmalemma expansion, depends on both intrinsic and extrinsic signals (Gallo and Letourneau, 2002, Tassew et al., 2014). These environmental cues influence axon growth and guidance by generating polarity, activating intracellular signals, and reorganizing membrane and cytoskeletal components.

Lipid rafts in specific regions of the GC aid in generating this polarity, stimulating axonal growth and serving as a lighthouse for axonal guidance. (Guirland et al., 2004, Kamiguchi, 2006) For example, lipid raft-associated transmembrane receptor Neogenin causes death or survival when Repulsive guidance molecule A is absent or present, respectively (Matsunaga and Chedotal, 2004). Blocking Neogenin-lipid raft association with various approaches enhances neuronal survival and promotes axonal regeneration in the injured adult optic nerve (ON) and spinal cord, demonstrating that modifying lipid rafts by regulating protein-lipid interactions (P:Ls) promotes axonal regeneration and functional recovery (Tassew et al., 2014).

With the advent of large-scale lipidomic, proteomic, and transcriptomic data integration, multi-omic analyses are possible and potential lipid-protein interactions are identifiable. However, multi-omic data acquisition for GC across development and during adult regeneration is lagging, with tools for visualization remaining underdeveloped, thereby hindering accessibility for end users and impeding biomedical research on devastating neurogenerative diseases. With this in mind, we have applied this multi-omic strategy to profile the GC proteome and lipidome at different developmental stages and regions, focusing on the GC plasmalemma and particulate fractions. In doing so, we characterize the unique lipidome and proteome of GCs across development. Integration of our GC data with lipidomic profiles of induced regeneration in the adult CNS has allowed us to identify functional changes in the lipid landscape that mark the transition into a growth-permissive state. We expect that with this work will provide an essential molecular resource for linking developmental and regenerative neurobiology and further expanding our knowledge on the enigmatic GC.

## Results

### Proteomic Profiling of GCs across Fraction and Developmental Stage

**Figure 1:**
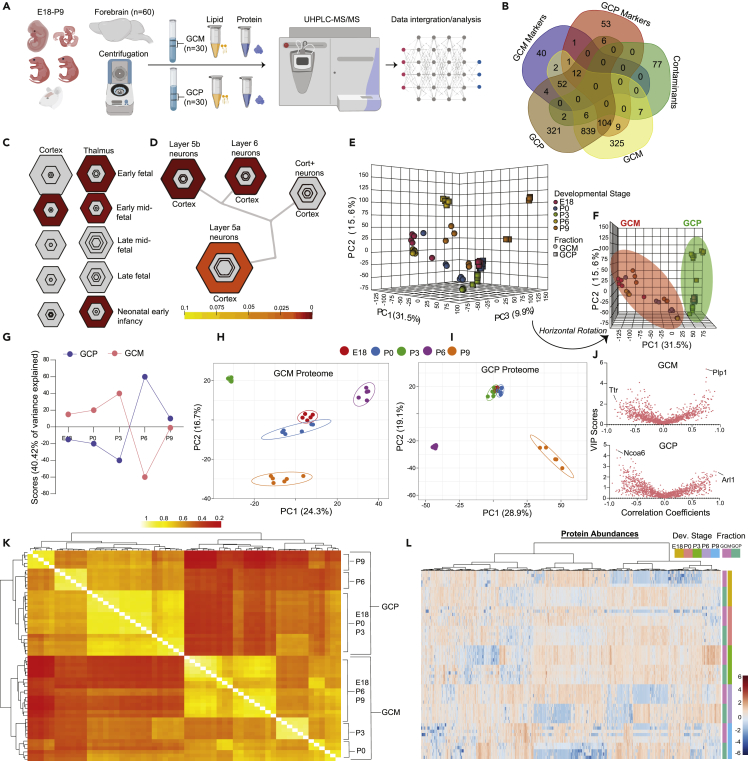
Proteomic Profiling in Growth Cones Across Developmental Stages (A) Study overview detailing the generation of proteomic and lipidomic data from the growth cone membrane (GCM) and growth cone particulate (GCP) across five developmental stages (E18, P0, P3, P6, and P9) in the C57BL/6 mouse model. For the growth cone proteome, there were six biological replicates (except E18, GCM, and P9 GCP, which were *n* = 5) at each developmental stage for generation of GCM (*n* = 29) and GCP (*n* = 29) samples (total *n* = 58). (B) Proteomic comparison in the GCM (*n* = 1,357 proteins) and GCP (*n* = 1,346 proteins) across age groups with previously published proteomic GC data (Estrada-Bernal et al., 2012, Nozumi et al., 2009, Igarashi, 2014). Proteins discovered in literature review are labeled as “GCM Markers” and “GCP Markers”; “Contaminants” include proteins that are found in much higher concentrations in the cytosol of non-growth cone cells. (C and D) Bull's-eye plots from a specific expression analysis on proteomics data for the examination of neuronal cell-specific and regional-specific enrichment across development in the mouse brain utilizing data collected from expression profiles of targeted cell types from bacTRAP mouse lines (Dougherty et al., 2010, Heintz, 2004, Xu et al., 2014). Colors are coded by p values (enrichment analysis), and varying stringencies for enrichment are represented by the size of the hexagon color; less specific, outer hexagons; more specific, center. There is notable enrichment (pSI < 0.05, p<0.01, pSI and p are specificity index and Fisher-exact test respectivity) of GC proteins in the cortex and thalamus brain regions in the early fetal, early-mid fetal, and neonatal-early infancy developmental stages (C) and in a cluster of neurons of layer 5a, layer 5b, and layer 6 in the cortex (D). (E) A 3D unsupervised principle-component analysis (PCA) of mouse growth cone proteome (*N* = 2,703) across developmental stage and fraction (GCM, GCP). (F) Horizontal rotation around the PCA x axis revealed complete separation of GCM and GCP in principle component 1 (PC1). (G) ANOVA-simultaneous component analysis resolving the interaction of developmental stage and fraction on growth cone proteome. (H and I) PCA of GCM proteome (H) and GCP proteome (I) across developmental stages revealed clustering of early developmental stages (E18 and P0) and separation of late developmental stages (P3, P6, and P9). (J) V-plots of variable importance in projection (VIP) scores (y axis) from partial least-squares regression and correlation coefficients (x axis) from Pearson correlation of proteins that correlated with a linear increase (positive value) or decrease (negative value) along developmental stage; GCM, top; GCP, bottom. (K) Correlation matrix (Pearson) of samples (*n* = 58); white/yellow indicates a higher correlation; red indicates a lower correlation. x and y axes represent Pearson coefficient and developmental age of GC fractions (as indicated), respectively. (L) Heatmap of growth cone proteome abundances across developmental stage and fraction; Euclidian distance measure and Ward clustering algorithm, features are autoscaled (red denotes positive, blue denotes negative). x and y axes represent relative protein abundance and developmental age and type of GC fractions (as indicated), respectively.

We performed high-performance liquid chromatography-tandem mass spectrometry (LC-MS/MS) analysis of the proteome and lipidome of GC from C57BL/6 mice across five age groups (E18, P0, P3, P6, and P9) from two GC fractions: growth cone membrane (GCM) (Ellis et al., 1985, Nozumi et al., 2009) and growth cone particulate (GCP) (Nozumi et al., 2009, Pfenninger et al., 1983) (Figure 1A). These fractions were generated through established differential centrifugation techniques (Ellis et al., 1985, Pfenninger et al., 1983) with lipid (Bligh and Dyer, 1959) and protein (Estrada-Bernal et al., 2012, Nozumi et al., 2009, Poulopoulos et al., 2019) extraction with enhanced offline fractionation before LC-MS/MS to increase coverage. Developmental time points were chosen based upon our understanding of brain development in the murine model. In mice cortex, E18 is characterized by extension or elongation of axons toward targets, whereas around P0-P3 is when axons start innervating targets and branching occurs (Catapano et al., 2001, Floeter and Jones, 1985). At P6-P7 is when target innervation is generally complete and a subpopulation of cortical neurons undergoes developmental cell death (Spreafico et al., 1995). By P9 GCs have mostly disappeared (Portera-Cailliau et al., 2003). We detected 1,357 quantifiable proteins in the GCM fraction and 1,346 in the GCP fraction across all developmental stages. The reproducibility of fractions was evaluated: coefficients of variation across developmental stages was less than 11%, and intra-age variation was less than inter-age variation in both the GCM and GCP fractions (Figures S1A and S1B; Table S1; normalized data in Figures S2A–S2E). Total proteomics data clustered 100% GCM and GCP samples by fraction and into their respective developmental stages (Figures S1C–S1E). This novel dataset serves as the first characterization of the combined GC proteome and lipidome across development and fraction (Figures S1A–S1H). We have developed an online visualization suite for the interrogation of this multi-omic dataset (https://gcinsights.herokuapp.com/).

Figure360. An Author Presentation of Figure 4

To assess the purity of our GC proteome, we compared our proteomics data with data in previously published reports (Ellis et al., 1985, Estrada-Bernal et al., 2012, Igarashi, 2014, Nozumi et al., 2009, Pfenninger et al., 1983), which we label “GCM Markers” and “GCP Markers.” We also compared our identified proteins with known contaminants, which include all proteins that are found in much higher concentrations in the cytosol of non-GC cells (Figure 1B and Table S2) (Estrada-Bernal et al., 2012). We have adopted previously published contaminant-characterizing criteria (Estrada-Bernal et al., 2012). We used dot blot GC fractions, regenerating ON, extracts of brain (positive control), immortalized human embryonic kidney (negative control) probed with antibodies to myelin basic protein (MBP) and myelin oligodendrocyte glycoprotein (MOG), and mass spectrometry to detect the presence and absence of selected proteins for validation (Figure S2F). We found only 14 and 9 contaminants in our GCM and GCP proteomes, respectively. There were 325 novel proteins in the GCM that were not previously characterized as GCM markers (not known contaminants, or were in the GCP). In addition, we discovered 321 novel proteins in the GCP. In the GCM, we discovered several endocytosis pathway-related proteins, including Rab4a, Rab5b, and Snx2. In the GCP, unique proteins included a number of proteins related to actin skeleton organization, such as Jam3, Fmnl2, and Twf2. The most abundant proteins in the GCM and GCP were Gap43, Tuba1a, Tubb2b, and Ncam1, and Actg1, Tubb3, Cltc, and Dpysl2, respectively. We also placed Fabp7 as a GCP marker consistent with a previously published report (Nozumi et al., 2009). We then performed a specific expression analysis on our GC proteomics data to determine the origin (neuronal cell types and brain regions) utilizing expression profiles of targeted cell types from bacTRAP mouse lines (GSE360068; GSE38668; GSE30626; GSE13379; GSE43164) (Dougherty et al., 2010, Heintz, 2004, Xu et al., 2014). We found significant enrichment (pSI <0.05, p < 0.01) of our proteins in the cortex and thalamus brain regions in the early fetal, early-mid fetal, and neonatal-early infancy developmental stages (Figure 1C). In further examination of the cell type-specific expression, we found that our GC proteins were significantly enriched (pSI <0.05, p < 0.05) in a cluster of neurons of layer 5a, layer 5b, and layer 6 in the cortex (Figure 1D). These results provide evidence for the purity and quality of our GC data.

A 3D unsupervised principle-component analysis (PCA) of the GC proteome revealed separation by fraction and across ages, with fraction fully separating based on PC1 and PC2 (Figures 1E and 1F). We subsequently ran an ANOVA-simultaneous component analysis to resolve the impact of age and fraction on the GC proteome. As expected, fraction explained 100% of the variation in PC1, whereas age explained 45% of the variation. In examining the interaction of age and fraction, the greatest variance occurred between P3 and P6, a developmental time point that characterizes the shift from branching to pruning processes (Figure 1G) (Shigeoka et al., 2016). In focusing on within-fraction age-based differences, we found that a 2D PCA explained 41% of the variance in the GCM and 48% of the variance in the GCP (Figures 1H and 1I). Correlation analysis between all samples showed that despite no significant positive correlations between the GCM and GCP, there were notable positive correlations within each fraction and between developmental stages (Figure 1K). A heatmap of peak intensity values across age/fraction confirms differential enrichment patterns across age, with the most notable changes between fractions (Figure 1L).

A main feature of GCs is the fine control of protein turnover during development and in response to external cues (Gallo and Letourneau, 2002). However, no work to date has fully characterized protein accretion in GCs across development. We determined the proteins whose abundances most positively and negatively correlate linearly along development (Figures S3A and S3B). Among identified GCM proteins, the ones most significantly positively correlated (p < 0.01, Pearson *r* > 0.7) with age were Thy1 and Syn1, whereas Ncam1 and L1cam were among the ones most negatively correlated (p < 0.01, Pearson *r* < -0.7). In the GCP, Aldoa and Tubb3 were the most positively and negatively correlated proteins with age, respectively (Figures S3C and S3D). To validate the GCM proteomic changes across development, we compared our findings with transcriptomic data from human (GSE13344 and GSE25219) and mouse (SRP031888) developmental databases for *Ncam1* and *Syn1*. We found that *Ncam1* peaked in early development in human across brain regions and then decreased postnatally in human and mouse, with higher expression localized to the cortical areas (Figures S4A–S4D). *Syn1* had an opposite age-based expression pattern than *Ncam1*, with a more disperse regional expression (Figures S4E–S4H). Recently available proteomics datasets (Kawasaki et al., 2018) from GC phosphoproteomics datasets are consistent (Figures S3E and S3F) with our overall GC proteomic analyses (although post-translational modified proteome is beyond the scope of current study).

We also ran a partial least squares regression on the GC proteomic data to identify subsets of proteins that most significantly predict developmental stage (R^2^ = 0.9993 and Q^2^ = 0.9588, accuracy: 0.9655). V-plots of variable importance in projections (VIP) scores in modeling of GC development against correlation coefficients revealed the most important predictors whose abundances change linearly across development (Figure 1J). Through this analysis, we found that the most significant GC proteins in the prediction of early and late developmental stages were related to regeneration, development, and lipid binding. For example, we identified that Ttr and Plp1 were enriched in E18 and P9 ages, respectively, and were important significant predictors of those developmental stages in regression (VIP>2). Ttr is a protein involved in the transport of retinol and has been shown to enhance nerve regeneration after crush, whereas Plp1, or proteolipid protein 1, is a major myelin-related protein with increasing evidence of expression in neurons (Fleming et al., 2007, Miller et al., 2009). Proteins of the proteolipid protein family have been recorded to be highly enriched on GCs (Luders et al., 2017, Mita et al., 2015). In the GCP, we found that Ncoa6 was a significant predictor of E18, whereas Arl1 was a predictor of P9. Ncoa6 is a hormone receptor localized to the cytosol and nucleus involved in cell survival and development, whose major ligands include fatty acids and cholesterol derivatives (Mahajan and Samuels, 2008). Arl1 is a cytosolic protein involved in positive regulation of phospholipase activity. These results have allowed us to identify intricate GC proteomic signatures and patterns at different biologically relevant time points and compartments.

### Proteomic Pathway Analysis

**Figure 2:**
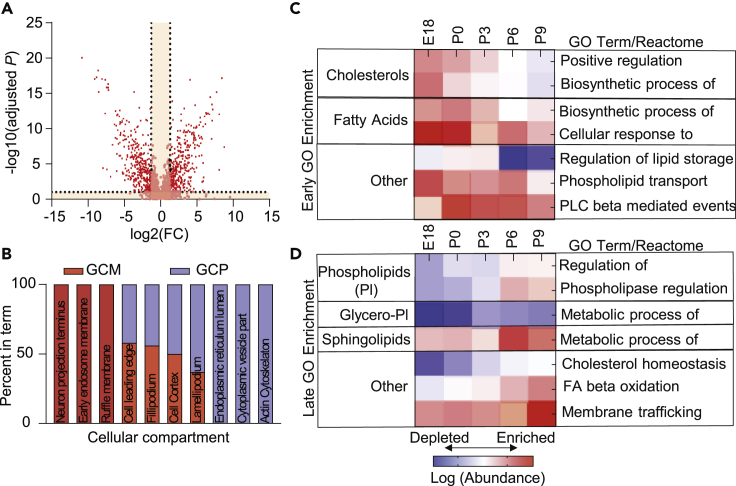
Growth Cone Proteome Pathway Analysis (A) Volcano plot of significantly different proteins between the growth cone membrane (GCM, right) and growth cone particulate (GCP, left). Proteins with significantly different abundances are denoted by red squares plot [FDR-adjusted p < 0.05, t-test, fold change > 2.0]. FDR, false-discovery rate. (B) Percentage of daughter Gene Ontology (GO) cellular compartment growth cone-related terms associated with the GCM-enriched (red) and GCP-enriched (blue) proteome. (C and D) Enriched lipid-related GO biological processes and reactome pathways (p < 0.05, enrichment analysis) in early (C) and late (D) developmental stages for growth cone proteome (*N* = 2,703) across development. Enrichment was analyzed by ClueGO; red denotes enrichment; blue denotes depletion.

We next aimed to characterize the molecular functions and subcellular components of the GC proteome. Analysis with molecular function terms revealed that proteins with “binding”-related functions were the most common in both the GCM and GCP, with no proteins detected with “transcription regulator activity” (Figures S5A and S5B). Cellular component enrichment was identified in ClueGO, and terms were subsequently grouped through clustering by semantic similarity measures (Supek et al., 2011). We identified distinct GC-related clusters in both the GCM and GCP, including “growth cone part,” “filopodium,” “lamellipodium,” and “actin-based cell projection” (Figures S5C and S5D), which corroborates our findings. To identify specific cellular components that differed between the GCM/GCP, we ran enrichment/depletion analysis on proteins whose abundances were significantly different, as identified in a volcano plot (false discovery rate [FDR]-adjusted p < 0.05, fold change > 2.0) (Figure 2A). We found that the GCM-only proteome was enriched for membrane-related terms, whereas the GCP-only was enriched in cytoskeleton and cytosolic terms (Figure 2B). In examination of the GC biological processes, we performed Gene Ontology (GO) analysis on proteins identified in the correlation analysis. With increasing age in the GCM and GCP, there was an elevation in processes related to localization, whereas decreasing age in the GCM and GCP showed an elevation in processes associated with development (Figure S6A). Focusing on lipid-related biological processes, we found that the components of pathways related to cholesterol and fatty acid biosynthesis were enriched in early development and decreased with age, highlighting the importance of these processes in axonal growth (Figures 2C and S6B) (Vance et al., 2006). We also observed a significant enrichment at P0 of lipid signaling through phospholipase C beta-mediated events. On the other hand, processes related to phospholipid, sphingolipid, and glycerophospholipid metabolism increased with age (Figures 2D and S6C). This analysis demonstrates enrichment of compartment-specific and stage-specific processes of GCs, including the regulation of lipid metabolic processes along different development stages. These results provide a foundation for better and further understanding of potential proteome-driven lipid class switching along development.

### The Growth Cone Lipidome

**Figure 3:**
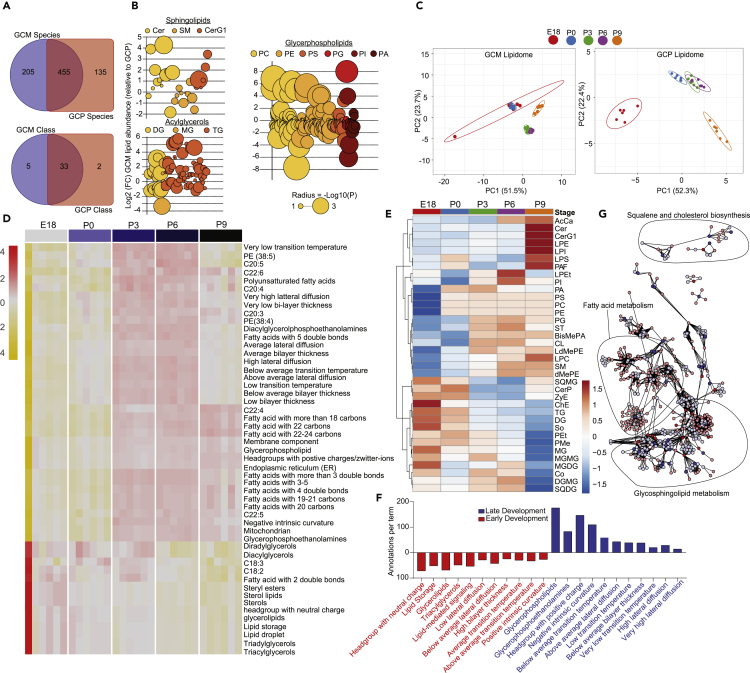
Lipidomic Profiling in Growth Cones Across Developmental Stages (A) Venn diagram comparing lipid species (top) and classes (bottom) between the GCM (*n* = 30 mice; *n* = 660 species; *n* = 38 classes) and GCP (*n* = 30 mice*; n* = 590 species; *n* = 35 classes). (B) Lipidomic analysis of GCM lipid classes in several lipid categories, as illustrated. Values are log2 fold change relative to GCP lipids with circles indicating a lipid species within a lipid class (color). Radius of the circle is coded by FDR-adjusted p value from t tests. (C) PCA across developmental stages; GCM lipidome, left; GCP lipidome, right. (D and E) Heatmap of enriched lipid ontology terms (LION) (D) and lipid class abundances (E) across developmental stages in the GCM. LION analysis is based on GCM lipid species. (F) Number of annotations (y axis, lipid species) per significantly enriched LION term (x axis; FDR-adjusted p < 0.05 enrichment analysis) in the GCM in early development (red; E18, P0) and late development (blue; P3, P6, P9). Early and late developmental groups were determined by a k-means clustering algorithm of GCM lipid species (*n* = 660) (Figure S8). FDR, false-discovery rate. (G) Protein-lipid (P:L) interaction network of GCM lipidome (*n* = 38 lipid classes) and total growth cone proteome; lipids are denoted in red; proteins, in blue. Metabolic pathways were identified by KEGG and are circled and labeled appropriately.

Untargeted lipidomics on the same GC samples quantified 660 lipid species across 38 lipid classes in the GCM. In the GCP, we quantified 590 lipid species across 35 lipid classes. We found 205 unique lipid species in the GCM and 135 unique species in the GCP (Figure 3A). Unique lipid classes in the GCM were cholesterol esters (ChE), ganglioside GD1a, phosphatidylinositol phosphates, sulfatides, and sulfoquinovosyl diacylglycerol, whereas in the GCP they were dihexosylceramides and lysophosphatidylglycerols. Coefficients of variation across developmental stages were less than 10%, and intra-age variation was less than inter-age variation in both the GCM and GCP (Figure S1B). Hierarchical clustering showed that 93% and 96% of the GCM and GCP samples, respectively, clustered into their age groups based on lipid data, whereas significant overlap of GCM and GCP samples was observed (Figures S1F–S1H).

An examination of lipid classes in the GCM and GCP revealed a strikingly similar pattern based on relative abundance, with phosphatidylcholines (PC), phosphatidylethanolamines (PE), and phosphatidylserines (PS) having the greatest abundances in both fractions (Figure S7A). A volcano plot of the common lipid classes in the GCM and GCP revealed that four lipid classes were significantly different; lysophosphatidylserines and cardiolipins (CL) were greater in the GCP, whereas monohexosylceramides (CerG1) and coenzymes were greater in the GCM (Figure S7B). We found several significantly enriched and depleted species that fell within sphingolipid, acylglycerol, and glycerophospholipid categories between the GCM and GCP (Figure 3B). Notably, we found a number of species in the sphingolipid (ceramides [Cer], sphingomyelin [SM], and CerG1) and acylglycerol (triacylglycerol [TG], monoacylglycerol [MG], and diacylglycerol [DG]) categories that were elevated in the GCM. In regard to the glycerophospholipids (PC, PE, PS, phosphatidylglycerol [PG], phosphatidylinositol [PI], phosphatidic acid [PA]), we found that PI and PA species tended to be elevated in the GCP, whereas a number of PE and PC species were elevated in the GCM.

A 2D PCA explained 75.2% and 74.7% of the variance in the GCM and GCP by age, respectively (Figure 3C). Correlation analysis of lipid classes showed that in the GCM, CerG1 and several lysolipid classes were significantly positively correlated with developmental stage, whereas the acylglycerols (MG, DG, and TG), sphingosines (So), ChE, and zymosterol esters (ZyE) were significantly negatively correlated with developmental stage (Figure S7C). In the GCP, correlation analysis of lipid classes showed a similar trend as the GCM. However, CL and ZyE were the most positively and negatively correlated with developmental stage, respectively (Figure S7D). We then clustered lipid classes using a Ward clustering algorithm and examined the abundance across developmental stage in both fractions. There was notable clustering of the acylglycerols, cholesterols and zymosterols in early development and lysolipid classes in late development (Figures 3E and S7E).

Given the diverse changes in lipid classes across development in the membrane of GCs, we next investigated the changes in the biophysical and chemical properties of the GCM lipidome by examining enrichment of lipid ontology (LION) terms (Clair et al., 2019, Molenaar et al., 2018). A heatmap of significantly enriched lipid ontologies across development showed a distinct separation between E18-P0 and P3-P9 age groups, with E18-P0 enriched for “fatty acids with 2 double bonds,” “sterol lipids,” and “lipid droplet” terms (Figure 3D). P3-P9 age groups were enriched in “fatty acids with more than 3 double bonds,” “membrane,” and “glycerophospholipids” LION terms. Considering the separation observed in the heatmap, we decided to run a k-mean clustering algorithm on GCM lipid species with a 2-cluster solution to observe sample grouping. Developmental stages grouped with E18 and P0 in one cluster and P3-P9 in another (Figures S8A and S8B). We subsequently labeled the E18-P0 cluster as “early development” and P3-P9 as “late development” and re-ran LION analysis focusing on biophysical properties. We observe significant enrichment of processes associated with “low lateral diffusion,” “high bilayer thickness,” “positive intrinsic curvature,” and “above average transition temperature” in early development (Figure 3F). Intriguingly, we observe a complete opposite pattern in late development, with significant enrichment in processes associated with “high lateral diffusion,” “below average bilayer thickness,” “negative intrinsic curvature,” and “below average transition temperature.” The aforementioned results demonstrate the biophysical and chemical properties of the GC plasmalemma that undergo dramatic stage-specific changes, which actively contribute to membrane organization and function.

### Growth Cone Bi-omic (Proteomic and Lipidomic) Correlation Analysis

**Figure 4:**
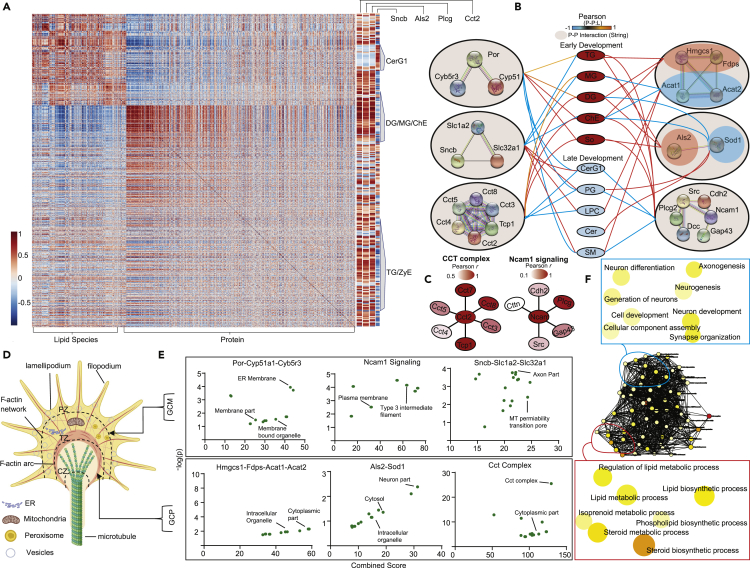
Protein:Lipid Correlations for Biological Insight For a Figure360 author presentation of this figure, see https://doi.org/10.1016/j.isci.2020.100836. (A) Correlation matrix (Pearson) between 660 GCM lipid species and 2,703 growth cone proteins. Zoomed-in plots on the right side of matrix detail correlations between a subset of individual proteins (columns) and GCM lipid species abundance (rows). CerG1, monohexosylceramide; ChE, cholesterol ester; DG, diacylglyceride; MG, monoacylglyceride; TG, triacylglyceride; ZyE, zymosterol. Positive values are red; negative values are blue. (B) Correlations between GCM lipid classes enriched in early (red) and late (blue) development and STRING protein-protein (PP) interactions. Colored lines outside interaction circle indicate the combined protein-lipid correlation coefficients for each protein in interaction as determined from averaging the transformed Fisher's *Z* values and back-transforming to Person *r* (*r*-to-*Z-*to-*r* transformation) (Methods); red denotes positive correlation; blue denotes a negative correlation. (C) Lipid-related CORUM protein complexes identified in panel (B). Colors indicate the correlations of each protein in complex with developmental stage. A darker red denotes a more negative correlation with developmental stage (i.e., decreases linearly with age). (D and E) Diagram of growth cone zones and putative organelle location (D) coupled with Jensen Compartments enrichment analysis using Fisher's exact test with Benjamini-Hochberg correction of lipid class-correlated interacting proteins (E) (top, GCM; bottom, GCP). pz, peripheral zone; TZ, transitional zone; CZ, central zone. (F) GO biological process network enrichment analysis of protein interactions identified in (B) highlighting processes related to lipid metabolism (bottom) and growth and development (top) (p < 0.05 enrichment analysis).

Lipid-related proteins were subsequently evaluated by examining P:Ls in MetScape and LipidMaps database. Integrating our GC proteomic and lipidomic data, we discovered a number of P:Ls that fell within “squalene and cholesterol biosynthesis,” “Fatty acid metabolism,” and “Glycosphingolipid metabolism” KEGG pathways (Figure 3G). Correlations between GCM proteomic and lipidomic datasets identified a subset of proteins that correlated with lipid species and class abundance in the membrane of GCs. Global correlation analysis revealed that, as a whole, proteomic and lipidomic data were not significantly correlated with each other. However, there were a number of individual proteins that significantly correlated with lipid species in specific lipid classes (Figure 4A). For example, Als2, Cct2, and Plcg2 positively correlated with cholesterol esters and MG, DG, and TG lipid species. Alternatively, Sncb correlated with CerG1 lipid species and negatively correlated with the acylglycerols.

P:Ls (Figure 3G), along with proteins identified in correlation (Figure 4A), were then probed for any protein-protein (P-P) interactions from STRING database. There were two requirements for our P:P analysis: all proteins (1) must be identified in our proteomics dataset (GCM or GCP) and (2) must significantly correlate with each other along development (positively or negatively). This analysis revealed a subset of interacting proteins that significantly correlate with early enriched and late enriched lipid classes in the GC membrane (Figure 4B). Of note, Por-Cyb5r3-Cyp51a1 positively correlated with TG, DG, ChE, and So lipid abundance. Fdps-Hmgcs1-Acat1-Acat2 and Als2-Sod1 were two sets of P:P interactions that had an opposite trend. Fdps-Hmgcs1 and Acat1-Acat2 were positively and negatively correlated with cholesterol esters, respectively, whereas Als2 was positively correlated with DGs and Sod1 was negatively correlated with DGs. We then integrated these data with CORUM to identify well-defined protein complexes that correlate with lipid abundance (Figure S9) (Giurgiu et al., 2019). We discovered proteins that compose the Cct complex (chaperon containing Tcp1; complex ID: 132) and Ncam1-Fgfr4 signaling complex (complex ID: 6480) all positively correlate with each other along development and GC membrane lipid class abundance (Figures 4B and 4C).

The subcellular localization and function of these P-P sets were probed utilizing data from Jensen Compartments database (Binder et al., 2014). We group P-Ps into the fractions from which they arose (GCM or GCP). Por-Cyb5r3-Cyp51a1, Ncam1 signaling complex, and Sncb-Slc1a2-Slc32a1 were from the GCM, whereas Hmgcs1-Fdps-Acat1-Acat2, Als2-Sod1, and the Cct complex were found in the GCP (Figures 4D and 4E). GCM P-Ps were predominately localized to the endoplasmic reticulum (ER) membrane, plasma membrane, and mitochondria (Figures 4D and 4E). GCP P-Ps were localized to the cytosol, intracellular organelles, and cytoplasm. To better elucidate the known roles of P-Ps, we analyzed the enriched GO biological process networks of these protein subsets (Figure 4F). We found that P-Ps were enriched in processes related to both development (i.e., “axonogenesis,” “neuron differentiation,” “neurogenesis”) and lipid metabolism (i.e., “lipid biosynthetic process,” “steroid biosynthetic process,” “phospholipid biosynthetic process”). These findings suggest evidence for the role of these proteins in fine-tuning lipid abundance in the GC membrane across developmental stages.

Another aim of our work was to identify possible molecules (both proteins and lipids) that could be important in supporting regeneration in the adult CNS. The role of these GC lipid-correlated proteins in axonal regeneration in adult is currently not known. To better elucidate the importance of these proteins in adult regeneration, we examined how their expressions change in response to knockouts of genes with important roles in axonal regeneration. We utilized knockouts of *Pten* and *Socs3* genes datasets for this analysis (GSE32309) (Sun et al., 2011). We discovered elevations in the expression of *Ncam1, Hmgcs1, Cct8, and Sod1*, with significant decreases in *Slc1a2*, in response to *Pten/Socs3* double knockout in the retinal ganglion cells of adult mice (Figure S10). These data indicate the importance of these proteins in both development and induced regeneration in the adult CNS.

### Common Lipids in Developing GCs and Adult Regenerating Optic Nerves

**Figure 5:**
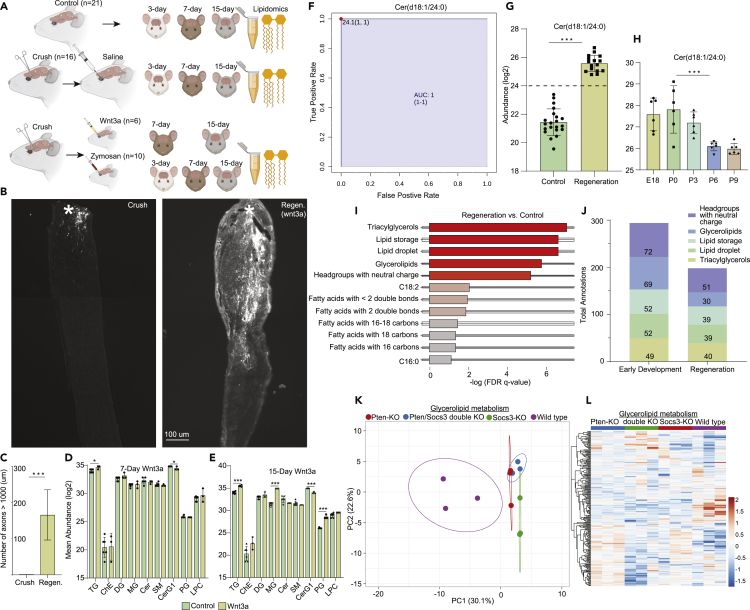
Common Lipids in Developing GCs and Adult Regenerating Optic Nerves (ON) (A) Study overview detailing the generation of lipidomic data from mouse normal ON (control, *n* = 21), crushed ON with intravitreal injection of saline (*n* = 16), and crushed ON with intravitreal injection of either Wnt3a (*n* = 6) or Zymosan (*n* = 10) across two to three time points post-treatment. (B) Cholera toxin B (CTB) staining to demonstrate Wnt3a-induced long-distance axon regeneration after ON crush compared with saline-injected as indicated. White star indicates crush site. (C) Axon regeneration distance count using CTB staining induced by Wnt3a compared with saline. Mean ± standard deviation (***p ≤ 0.001 regeneration compared with crush control, t-test). (D and E) Average lipid abundance of nine lipid classes between control and 7-day (D) and 15-day (E) post-Wnt3a injection; *p < 0.05; ***p < 0.001 (experimental compared to controls, t-test). (F) ROC of Cer(d18:1/24:0) for distinguishing ON regeneration from control; the sensitivity is on the y axis, and the specificity is on the x axis. The area under the curve (AUC) is in blue (AUC:1). (G and H) Average lipid abundance of Cer(d18:1/24:0) in adult ON regeneration compared with control (G) and across developmental stages in growth cone (H). Cer(d18:1/24:0) was highly significantly enriched in regenerating ON (Zymosan + Wnt3a, *n* = 16) compared with control (*n* = 21) and in growth cones in early development (*n* = 30); ***p < 0.001 [experimental compared with controls or in early development [P0] as indicated, t-test (G); ANOVA (H)]. (I) Enriched lipid ontology (LION) biophysical and chemical properties between batch-corrected regenerating (*n* = 16) and control (*n* = 21) ON lipid species (*n* = 208). (J) Number of annotations per lipid ontology term in early development in growth cone (E18,P0) and regeneration in adult optic nerve axons (Wnt3a + Zymosan). (K and L) PCA (K) and heatmap (L) of transcriptome across three genetic models of ON regeneration compared with wild-type focusing on genes in the glycerolipid metabolic process (GO:0046486) (*n* = 244 transcripts).

The assembly of new GCs is a requirement for the regeneration of axons after injury (Bradke et al., 2012). However, axons in the adult mammalian CNS, such as the ON, do not regrow, whereas axons of the PNS do regenerate. Successful regeneration and the formation of new GCs can be induced in the CNS through genetic modulation and therapeutic interventions. Despite notable mechanistic differences between axonal *de novo* growth in development and induced regeneration in adult in the CNS (Liu and Snider, 2001), one commonality is that the plasma membrane seems to expand in basically the same manner (Pfenninger, 2009). In regenerating axons of the CNS, the source of this new membrane is supplied by Golgi-derived anterogradely transported vesicles (Erez et al., 2007). Thus, we hypothesize the possibility of a set of lipid species or classes that may identify the non-regeneration to regeneration transition in adult, with the hope of improving our understanding of induced axonal regrowth in the CNS and uncover possible treatments to improve GC regeneration. We compared the lipidome of two models of adult axonal CNS regeneration (Wnt3a- and Zymosan-induced ON regeneration) (Chen et al., 2009, Patel et al., 2017, Yin et al., 2003) to the GCM lipidome enriched in early development in search of a common set of lipid species or classes. Briefly, ONs of 2-month-old mice were crushed ∼1 mm behind the globe and subsequently injected with saline, Wnt3a, or Zymosan intravitreally (Figure 5A). Lipids were extracted from mouse ON at 3, 7, or 15 days post injections and analyzed in the same manner as GCs, discussed previously. The 15-day post-Wnt3a-injection, cholera toxin B (CTB)-labeled axons showed an increased number of axons past the crush region, indicating an increase in the number of GCs and an active state of regeneration (Figures 5B and 5C).

Before analyzing the lipidome between control and regeneration, we aimed to determine if ON crush altered the ON lipidome or proteome in a significant way. To do this, we examined the lipidome and proteome between crush (with saline injection) and control (no crush) through an unsupervised PCA and t tests (FDR-adjusted p < 0.05) (Figures S11A–S11D). The PCA showed significant overlap, and there were no significantly different features in the proteome between control and crush. There were, however, 9 lipid species that were significantly lower in abundance in crushed ON compared with control (Figure S11A). These species were predominantly from the PC lipid class, with one species from the PE lipid class. Lipidomic profiling of 7-day post-Wnt3a administration revealed that only two lipid classes were slightly significantly different between control and Wnt3a regenerating ONs: CerG1 and TG (Figure 5D). CerG1 lipids were higher in control ON (p < 0.05), whereas TG lipids were higher in regeneration (p < 0.05). Interestingly, 15-day post-Wnt3a the magnitude of the difference in the TGs and CerG1s between control and regeneration rose drastically (p < 0.0001), with the addition of two significantly different lipid classes, MG and PG (Figure 5E). Both MG and PG lipids were found to be significantly greater in regenerating ON (p < 0.0001).

We subsequently combined Wnt3a and Zymosan lipidomic datasets to find common lipid species and trends that differentiate regenerating ONs from control. Data were normalized followed by ComBat batch correction (Figures S12A and S12B) (Goh et al., 2017, Johnson et al., 2007, Muller et al., 2016). Classical receiving operator characteristic (ROC) curves were employed to identify lipid species with the greatest ability to detect regeneration. The best classifier, based on area under the ROC curve (AUC), was Cer(d18:1/24:0) (AUC:1) (Figure 5F). Cer(d18:1/24:0) was significantly higher in regenerating ON (p << 0.0001) with the ability to place 100% samples into the correct group (Figures 5G and S12). To our surprise, despite the Cer lipid class as a whole being significantly elevated in P6 and P9 in GCs, the Cer(d18:1/24:0) species was significantly elevated in E18, P0, and P3 (p < 0.0001) (Figure 5H). For the analysis of global lipid trends (biophysical and chemical properties), we ran LION analysis comparing regeneration and control groups (Figure 5I). In comparing common significant lipid ontologies in regeneration in adult ON with early development (E18,P0) in GC, we discovered significant elevations in the number of annotations per term in “triacylglycerol,” “lipid storage,” “lipid droplet,” “glycerolipid,” and “headgroups with neutral charge” terms in both models (Figure 5J).

Owing to the observed significant changes in glycerolipid metabolism, which includes the metabolism of the acylglycerols and lysolipids, in both early development and regeneration, we analyzed transcriptomic changes in glycerolipid metabolism across three genetic models of ON regeneration: *Socs3*^(−/−)^, *Pten*^(−/−)^, and *Pten*/*Socs3* double knockout (Sun et al., 2011). Co-deletion of *Socs3*^(−/−)^ and *Pten*^(−/−)^ has been showed to result in axon regeneration in the ON. We identified 244 transcripts (from the GEO deposited data of our previously published article, Sun et al., 2011) that fell within the glycerolipid metabolic process (GO:0046486). An unsupervised PCA revealed a separation between wild-type and our three genetic models of regeneration, with a clustering of the models (Figure 5K). A heatmap of transcripts in glycerolipid metabolism showed differential expression of genes across models (Figure 5L). These findings provide evidence for the existence of a set of common lipid pathways and species that characterize the “growth non-permissive” to “growth permissive” transition in adult axons and in GC membranes in early development.

## Discussion

We have generated a multi-omic resource that integrates quantitative lipidomic and proteomic data from mammalian lipid GC membranes and particulate in replicate animals across five developmental stages. We have leveraged this multi-omic dataset to interrogate knowledge gaps in GC metabolism, membrane expansion, and molecular variability across development. GCs play a central role in the expansion of a neuronal processes leading edge in both development and adult regeneration through ruffling, or the process of membrane extension and retraction involving the formation of *lamellipodia* and *filopodia.* A GC's leading edge requires that new plasma membrane be continually laid down as it makes its way toward a target (Levitan and Kaczmarek, 2015). At the central core of the GC, there is an enrichment in mitochondria, ER, and vesicular structures, which are used to generate energy and molecules that aid in growth (Figure 4D). In late development stages, the extrinsic and intrinsic molecular milieu of GCs changes so that they are unable to grow as effectively toward their targets (Liu et al., 2011). Despite the vital role that these structures play in development, GC molecular changes at different developmental time points have hitherto been uncharacterized. In particular, the GC lipidome, a central player in plasma membrane extension and the maintenance of transmembrane protein levels, was previously undefined.

In addition, we have for the first time combined developmental and regenerative neurobiological lipidomic data to find common lipid trends, species, and processes that define the growth-permissive state. Developmental neurobiology focuses on the processes and mechanisms by which “cell number, position, shape, and patterns of connectivity are set during embryonic and early postnatal life” in the nervous system (Levitan and Kaczmarek, 2015), whereas regenerative neurobiology aims to determine how damaged neurons and their connectivity can be repaired or restored in adults (Tsonis, 2002). It is plausible that molecules that are important in development may be expressed again in mature cells to achieve repair and regeneration (Tomassy et al., 2010). A challenge in regenerative neurobiology is to understand the constant alteration of P:Ls during development that are pertinent and important for evoking adult neuroregeneration. This understanding is key for repair in traumatic damages to spinal cord and the ON, in diseases such as Parkinson disease and multiple sclerosis. High-throughput multi-omic analysis has become central to addressing these challenges.

Our proteomic enrichment analyses suggest that the GCM is enriched in neuron projection terminus, early endosome membranes, cells' leading edge, and filipodium cellular component terms, whereas the GCP is enriched in actin cytoskeleton, cytoplasmic vesicle, and ER lumen components (Figure 2B), consistent with rapid cytoskeleton dynamics (Blanquie and Bradke, 2018) and GC cytoskeletal remodeling during axonal regeneration (Hur et al., 2012).

Proteolipid protein family has been recorded to be highly enriched on GCs (Luders et al., 2017, Mita et al., 2015). Our finding of Plp1 enrichment is inconsistent with myelin contamination: (1) most abundant myelin components (for example, myelin basic protein [MBP]) undergo robust proteolysis and are ionized efficiently, yet they were not captured in our high-resolution mass spectrometry (Figure S2F, bottom panel); (2) our findings corroborated when probed for MBP and MOG in dot blots (Figure S2F); and (3) the finding of Plp1 and lack of other myelin components are thus suggestive of lack of myelin contamination. They are rather indicative of Plp1 expression in neurons as observed in earlier reports and found in GCs (Campagnoni and Skoff, 2001, Sarret et al., 2010, Timsit et al., 1992). Myelination occurs relatively late in mouse development in a defined temporal order. Myelination begins at birth in the spinal cord in mice and is almost completed at P60 in most brain regions (Baumann and Pham-Dinh, 2001). Taken together our data are consistent with innate GC presence of Plp1 at a time point (E18) when myelination has not even begun and suggestive of same intrinsic production and enrichment at late developmental stages rather a contamination, which would then have shown all other highly proteolysis-prone and ionization myelin proteins rather than the presence and enrichment of Plp1.

Early-stage GO enrichment of GC proteome showed enriched biosynthesis of cholesterols and fatty acids in E18 and P0, whereas late GO enrichment showed opposite trends for phospholipids, glycerophospholipids, and sphingolipids (Figure 2C). Lipidomic analysis confirmed GC stage-specific proteomic changes, detailing enrichment of cholesterols, acylglycerols (MG, DG, and TG), C18:3, C18:2, and fatty acids with two double bonds in early development, with enrichment of glycerophospholipids and glycerophosphoethanolamines in late development (P3, P6, P9) (Figures 3D, 3E, and 3G). The metabolic processes related to the positive regulation of cholesterol biosynthesis are enriched in early developmental stages, specifically E18 and P0. Prior reports suggest that brain *de novo* biosynthesizes all the cholesterol it requires (Edmond et al., 1991). Current experimental evidence is consistent with *de novo* synthesis of all or majority of cholesterol in the retina, eye (Lin et al., 2016), and brain (Cunnane et al., 2001). Our observation of positive regulation of cholesterol biosynthesis by enrichment of elements should be taken with the caveat that we cannot completely rule out contribution due to glial cells. However, the gliogenesis starts around E18 (Gallo and Deneen, 2014, Guerout et al., 2014, Rowitch and Kriegstein, 2010) and would be inconsistent with our findings. Our analysis is also inconsistent with enrichment of glial lipid signature; for example, arachidonic acid produced by astrocytes is not enriched in our analysis. This observed regulation of the lipid landscape along development aligned with lipid-induced changes of the plasmalemma properties, such as bilayer thickness, transition temperature, and membrane curvature (Holthuis and Menon, 2014). The early development is associated with enrichment of lipids associated with “low lateral diffusion,” “high bilayer thickness,” “positive intrinsic curvature,” and “above average transition temperature” (Figure 3F), in contrast to a complete opposite pattern (such as enrichment of lipids associated with high lateral diffusion, low bilayer thickness, negative intrinsic curvature, and below average transition temperature processes) in late development. Axonal/neurite growth is an active expansion of the plasma membrane. At later developmental time points, GCs undergo a change in function, from elongation to collateral sprouting. Currently, not much is known about the specific changes that occur in the membrane properties in GCs at varying biologically developmental time points. These findings are unique as they detail *in vivo* changes in lipids related to membrane properties in GCs along development. We believe that elevations in bilayer thickness during early developmental stages are, in part, a reflection of active insertion of plasmalemmal precursor vesicles. Our data suggest that these changes are also a reflection of underlying lipid switching in the plasma membrane. The identification of membrane biophysical changes along development are an essential first step garnering a more thorough understanding of plasma membrane expansion.

Furthermore, we identified a number of P:Ls that fell within the squalene and cholesterol biosynthesis and fatty acid and glycosphingolipid metabolic processes (Figure 3G). Lipid species and protein correlation analysis showed that Als2, Plcg2, and Cct2 significantly correlated with the abundance of cholesterol and acylglycerol lipid species in the GC membrane, whereas Sncb correlated with the abundance of monohexosylceramides (Figure 4A). As noted earlier, we observe the abundance of cholesterols from the same GC samples; we see that cholesterol is highest at E18 and P0 and decreases in abundance from P3 to P9. The relative abundance of proteins within this metabolic pathway decreases as the mouse ages from P3 to P9. Thus the lipidomic changes are aligned with GC stage-specific proteomic changes, and as stated above, inconsistent with contribution from gliogenesis. Correlating STRING P:P interactions with lipid classes enriched in early and late development revealed a number of novel networks with important roles in fine-tuning the abundance of lipid classes along development in GCs. Por-Cyp51a1-Cyb5r3, Ncam1 signaling complex, and Sncb-Slc1a2-Slc32a1 in the GCM and Hmgcs1-Fdps-Acat1- Acat2, Als2-Sod1, and Cct complex in the GCP showed significant alterations during early versus late development stages (Figures 4B–4E). Ncam1, Sncb, Slc1a2, Als2, and Sod1 have independently been shown to be associated with axonal growth or GC formation. However, our analysis has uncovered additional protein complexes and interactions that have yet to be identified in the GC.

We found enrichment of MG, DG, and TG lipid species as well as that of cholesterol during early development (Figures 4A and 4B). Prior studies investigating changes in membrane lipids during the period when nerve GCs become synapses are consistent with our findings (Martin and Bazan, 1992). The Por and Cyp51 protein complex was enriched in early developmental stages (Figures 4A, 4B, and 4E) and positively correlated with cholesterols and the acylglycerols (Figure 4B). The Por-Cyp51 complex is also known as lanosterol 14-alpha-demethylase protein complex. This post-lanosterol enzymatic conversion complex of the cholesterol biosynthetic pathway is composed of a cytochrome P450 enzyme CYP51 and its redox partner NADPH cytochrome P450 reductase. This complex has been shown to play a role in gametogenesis characterized by rapid membrane formation and extension (Rozman et al., 2002). Our findings suggest the importance of this complex in the regulation of cholesterol during axonal extension and elongation.

We also observed that transition from early to late development is concomitant with tremendous transition in lipid species with Cer, lysophophatidylcholines, and SMs emerging in greater amounts during late development (Figure 4B). Lipid composition changes render membranes more ordered or more disordered; the lateral and rotational freedom of molecules is reduced in more ordered membranes when compared with less ordered membranes where they are relatively more mobile. SMs induced changes in nanodomains, and also increase the miscibility for other lipids. Most of our understanding of nanodomains, curvature, miscibility, and phase transition of lipids is currently based on model membranes rather than real cellular membranes (Cebecauer et al., 2018). Thus, our understanding pertaining to composition changes related to changes in properties such as curvature and membrane elongation is rather limited for cellular or organelle membranes (Cebecauer et al., 2018, Ernst et al., 2016).

Cholesterol provides order in the membranes; however, with model membranes it has been learned that excessive cholesterol is inhibitory to embedding of membrane proteins (Cebecauer et al., 2018, Jerusalem et al., 2019). During early development in GCs where membrane extension is rapid, we have found cholesterol enrichment (Figure 4B). Cers enriched during late development stages are relatively rigid lipid molecules with a small head group and are known to form highly condensed monolayers and may help lock the membranes in place during late development. The Cer inclusion in lipid membranes increases lipid ordering and promotes solid/fluid-phase separation in the absence of cholesterol that may be applicable to some nanodomains (Cebecauer et al., 2018). Significant changes in lipid composition had bearing for curvature of membranes, important for GC turning and propagation as well as for transmembrane protein embedding. Various lipid species have different propensity to accumulate in curved areas or even to induce curvature. Curvature can be induced by the aggregation of lipids, and this curvature can affect lipid demixing. The model membrane and studies of biological membranes suggest that interleaflet coupling and communication in membranes is important during rapid membrane expansion and turning (Cebecauer et al., 2018), which are important during early development. Conversely, during late development, other changes including steps toward innervation are important, which is also expected to be associated with significant changes in lipids and membrane-embedded protein complexes.

We found Als2, CCT, and Plcg2 complexes positively correlated with cholesterol esters and MG, DG, and TG lipid species and negatively correlated with CerG1 lipid species (Figures 4B and 4E). Alsin or Als2 signaling is necessary for control of axonal growth (Jacquier et al., 2006). Conversely, Als2 deficiency results in axonal growth defects (Gautam et al., 2016, Otomo et al., 2008) and distal axonopathy (Deng et al., 2007). The GO biological processes, based on homology implicated Als2 and Hmgcs1 (cytoplasmic hydroxymethylglutaryl-CoA synthase) in axonogenesis. Plcg2 has been implicated in Wnt signaling. Plcg2 is also related to cell migration and thus is indirectly associated with membrane extension. Ovarian steroids increase glutamatergic-related gene expression including the glutamate transporter 2 (Slc1a2) in serotonin neurons of macaques (Bethea and Reddy, 2012). Slc1a2 complex is expressed in the nerve terminals of multiple brain regions (Zhou et al., 2019). Components of glutamate neurotransmission including Slc1a2 complex have been shown to be associated with dendritic spine proliferation and their stabilization (Bethea and Reddy, 2012). CCT complex ( chaperonin-containing TCP-1/TCP-1/TRiC complex) regulates transport of vesicles to the cilia contributing to ciliogenesis (Seo et al., 2010). CCT complex may be involved in transport of vesicles in GC plasmalemma extension. This complex is also known for restricting neuropathogenic protein aggregation by inhibiting autophagy and assisting in protein folding (Pavel et al., 2016), which may be necessary during early development. The Ncam1 complex has been shown to play critical role in precursor migration, ganglion cell aggregation, and neurite fasciculation necessary to form the enteric nervous system (Fu et al., 2006). It is likely that all these complexes are associated with neuritogenesis, various steps of GC membrane or plasmalemma expansion, and axonogensis. P:Ps were enriched in processes related to development such as axonogenesis, neuron differentiation, and neurogenesis as well as processes related to lipid metabolism such as lipid biosynthetic process, steroid biosynthetic process, and phospholipid biosynthetic process (Figure 4F). These novel networks are likely involved in fine-tuning lipid abundance in the GC membrane across developmental stages and in elongation of the membranes resulting in axon/neurite growth. They may also have overlapping roles. Thus, multi-omic analysis is a powerful approach to identify additional proteins and P:P and protein-lipid complexes that enable formulation of new testable hypotheses.

In examining the lipidome of induced axonal regeneration in the CNS, we uncovered similar patterns that aligned with the lipidome of early developmental GCs. For example, C18:2 and fatty acids with two double bonds showed significant alteration in crush versus long-distance regeneration (Figure 5F) in parallel to that in E18-P0-P3 (Figure 3D). In regenerating ON axons, our analysis shows increased Cer(d18:1/24:0), which is also elevated in the GC formation phase and undergoes a decrease during GC collapse (Figure 5H). In the trigeminal nerve ganglion selective neuronal degeneration is induced by doxorubicin (Bigotte and Olsson, 1987), which has now shown to result in concomitant decreased abundance of Cer(d18:1/24:0) (Snider et al., 2019). Conversely, maprotiline, an antidepressant drug, has been shown to induce regeneration of noradrenergic axon terminals and significant increases in the pre-frontal cortex of Cer(d18:1/24:0) (Lee et al., 2009). Membrane leaflet dynamics studies from other fields suggest that Cer(d18:1/24:0) may influence cytoskeleton domains and progression of GCs, opening up a new avenue for investigation. Our analysis thus provides additional leads, molecular complexes, and segments of pathways that affect human health and progressive neurodegenerative disease and trauma-induced degeneration. Our visualization may aid in interrogation of such protein and lipid pathways and potential interactions for others to generate their own hypotheses.

### Limitation of the Study

• Our study isolated GCs from the brain using established preparation methods. The GCs of the ON may differ in the composition from those of other components in the CNS.
• The established method of GC preparation is enrichment and not absolute purification. Thus, the preparation is not contamination free and the contamination heterogeneity cannot be independently verified for each replicate.
• The accuracy in the identification of the lipids and the confidence in the relative abundance levels are limited by current instruments and methods.

## Methods

All methods can be found in the accompanying Transparent Methods supplemental file.

## Data and Code Availability

All proteomics data associated with the article are being deposited in PRIDE proteomeXchange (http://www.proteomexchange.org/) (accession IDs PXD012134 and PXD016197). Transcriptomic expression data used are deposited at the Gene Expression Omnibus (GEO) (accession numbers: GSE32309, GSE360068; GSE38668; GSE30626; GSE13379; GSE43164, GSE13344 and GSE25219, SRP031888). All lipidomics data will be available at the Metabolomics Workbench, http://www.metabolomicsworkbench.org (Project IDs: PR000856). Processed growth cone proteomic and lipidomic data can be accessed at the following open access web utility: https://gcinsights.herokuapp.com/. Any additional data may be made available upon reasonable request to the corresponding author.

## Supplemental Information

Document S1. Transparent Methods, Figures S1–S12, and Table S1

Table S2. List of Growth Cone Known Proteomic Markers, Contaminants, and New Findings, Related to Figure 1

Data S1. Growth Cone Proteome across Ages, Related to Figure 1

Data S2. Growth Cone protein Pathway, Related to Figure 2

Data S3. Growth Cone Lipidome across Ages, Related to Figure 3

Data S4. Protein-Lipid Correlations, Related to Figure 4

Data S5. Common Lipids in Growth Cone and Regenerating Neurons, Related to Figure 5
