# Supplementary material for: Multi-Omic Analyses of Growth Cones at Different Developmental Stages Provides Insight into Pathways in Adult Neuroregeneration
Source: iScience. 2020 Jan 14;23(2):100836. doi: 10.1016/j.isci.2020.100836 (PMC6997871; doi:10.1016/j.isci.2020.100836)
Supplement: Document S1. Transparent Methods, Figures S1–S12, and Table S1 [file mmc1.pdf]

iScience, Volume *23*

Supplemental Information

Multi-Omic Analyses of Growth Cones at Different Developmental Stages Provides Insight into Pathways in Adult Neuroregeneration

Muhammad Zain Chauhan, Jennifer Arcuri, Kevin K. Park, Maroof Khan Zafar, Rabeet Fatmi, Abigail S. Hackam, Yuqin Yin, Larry Benowitz, Jeffrey L. Goldberg, Mohammad Samarah, and Sanjoy K. Bhattacharya

Supplemental Information

Multi-omic analyses of growth cones at different developmental stages provides insight into pathways in adult neuro-regeneration

Muhammad Zain Chauhan[1,2], Jennifer Arcuri[1, 2,3], Kevin K. Park[2, 3,4], Maroof Khan Zafar[5], Rabeet Fatmi[6], Abigail S. Hackam[1,2,3], Yuqin Yin[7,9], Larry Benowitz[8,9], Jeffrey L. Goldberg[10], Mohammad Samarah[6], Sanjoy K. Bhattacharya[1, 2,3]

1. Bascom Palmer Eye Institute, University of Miami, Miami, FL, 33136
2. Miami Integrative Metabolomics Research Center, University of Miami, Miami, FL, 33136
3. Neuroscience Graduate Program, University of Miami, Miami, FL, 33136
4. Miami Project to Cure Paralysis, University of Miami, Miami, FL, 33136
5. Department of Biochemistry and Molecular Biology, University of Arkansas for Medical Sciences, Little Rock, AR 72205
6. Department of Computer Science, Florida Polytechnic University, Lakeland, FL 33805
7. Department of Neurosurgery, Harvard Medical School, Boston, MA 02115
8. Department of Ophthalmology, Harvard Medical School, Boston, MA 02115
9. Department of Neurosurgery and F.M. Kirby Neurobiology Center, Boston Children's Hospital, Boston, MA 02115
10. Department of Ophthalmology, Stanford University School of Medicine, Stanford, CA 94305

This document contains Transparent Methods, Supplemental References and Figures S1-S12, Supplemental Table S1 (list of samples).

Supplemental Table S2 (List of growth cone known proteomic markers, contaminants, and new findings) is provided as an excel file.

Transparent Methods

Data reporting. Samples for all MS-based analysis were randomized prior to data acquisition. To estimate an appropriate group size for this study, statistical analysis was performed incorporating our preliminary data employing SAS/STAT software in consultation with our Biostatistical core experts. The following assumptions were made: that the tissue samples are independent for each protein/lipid, that equal sample sizes exists between comparative groups and that the proportion of a given protein/lipid being present in embryonic stages may or may not be equal to that in the postnatal group. The proportion of proteins/lipids that could be detected in embryonic and postnatal developmental stages are based on our preliminary data. The minimum number of animals needed per group for coverage of 100% in embryonic stages (concomitant with 83.3% proteins in postnatal stages) to achieve 80% power (type I error or false positive rate 0.05) and 90% power (type I error rate at 0.01) are 4 and 6, respectively. With these group sizes we expect to cover 83-100% proteins/lipids across developmental stages covering over 95% unique proteins/lipids. Similar size determination was made for control, crush, and regeneration ON groups in adult neuroregenerative studies. Animals chosen to receive treatment (i.e., Wnt3a, Zymosan) for in vivo experiments were chosen randomly.

Growth cone and ON regeneration mice. All animal procedures were performed in accordance with the ARVO Statement for the Use of Animals in Ophthalmic and Vision Research and under the University of Miami Institutional Animal Care & Use Committee (IACUC) approved protocols. All experiments described here conform to the relevant regulatory standards. Mice utilized in this study were sourced from The Jackson Laboratory and were subsequently bred and housed at the McKnight 3rd floor vivarium under AAALAC (https://www.aaalac.org/) approved standard conditions and diet with 12 hours light-dark cycles. Animals were euthanized using carbon dioxide exsanguination following AAALAC standard methods at the endpoints.

ONC, intravitreal injections, and axon quantification. The optic nerve crush, intravitreal injections and axon quantification followed established procedures as in our prior published studies (Park et al., 2010; (Park et al., 2008; (Patel et al., 2017; (Trzeciecka et al., 2019).

Growth cone sample collection and differential fractionation. All animal procedures were performed in accordance with the ARVO Statement for the Use of Animals in Ophthalmic and Vision Research and policies of the University of Miami Institutional Animal Care & Use Committee (IACUC). We carried out lipid and protein profiling of growth cone membrane (GCM) and growth cone particulate (GCP) fractions from forebrains of E18, P0, P3, P6, and P9 C57BL6/J mice fractionated by sucrose gradient ultracentrifugation as described in previously published reports. (Ellis et al., 1985; (Estrada-Bernal et al., 2012; (Pfenninger et al., 1983)

Lipid profiling. Lipids from each fraction were extracted using chloroform, methanol and water mixture to obtain phase separation. The extracted lipids corresponding to 100 µg proteins were used for untargeted liquid chromatography Q-Exactive Orbitrap tandem mass spectrometry (LC-MS/MS) for profiling. All solvents were LC-MS/MS grade. Methanol (6 ml) and chloroform (3 ml) were added to each sample (corresponding to 100 µg of total protein from each fraction GCM or GCP). After 2 min vigorous vortexing and 2 min sonication in an ultrasonic bath, the samples were incubated at 48˚C overnight (in borosilicate glass vials, PTFE-lined caps). The following day, 3 ml of water (LC-MS grade) and 1.5 ml of chloroform were added, samples were vigorously vortexed for 2 min and centrifuged at 3000 RCF, 4˚C for 15 min to obtain phase

separation. Lower phases were collected. Remaining samples (upper and interphases) were re-extracted by addition of 4.5 mL of chloroform and centrifugation at 3000 RCF, 4˚C for 15 min to obtain phase separation. Lower phases from both extractions were combined and dried in a centrifugal vacuum concentrator. Samples were stored at -20˚C and were reconstituted in 150 µL of chloroform:methanol (1:1) before mass spectrometric analysis.

Protein profiling. In parallel to lipids, protein samples from each differential centrifugation fraction were reduced, alkylated and digested using trypsin and Lys-C proteases, followed by LC-MS/MS. Proteome Discoverer 2.2 was used for bioinformatics analysis. Protein concentrations were determined using BCA assay (Thermo Fisher Scientific, Waltham, MA), according to manufacturer's instruction. Approximately 100 µg of proteins were precipitated with four volumes of ice-cold acetone overnight at -20˚C, proteins were subsequently centrifuged (15 min, 4˚C, 18,000 RCF) and pellets were resuspended in 20 µl of 0.2% ProteaseMAX (Promega)/50 mM ammonium bicarbonate (ABC; 50 mM in a final volume of 93.5 µl). Proteins were reduced with dithiothreitol (DTT): 1 µl of 0.5 M (final concentration ~5 mM) for 20 min at 56˚C. Next, proteins were alkylated using iodoacetamide (IAA): 2.7 µL of 0.55 M (final concentration ~15 mM) for 15 min at room temperature in the dark. 1 µl of 1% ProteaseMAX/50 mM ABC with 4 µg of Trypsin/Lys-C mix (Promega; 1:25 trypsin-to-protein ratio) was added. Digestion was carried out in a total volume of 100 µl (1µg of total protein/µl) for 4 h at 37˚C. Samples volumes were then adjusted to 300 µl with 0.1% trifluoroacetic acid/50 mM ABC. Pierce High pH reversed-phase fractionation Kit (Thermo) was used to separate peptides into eight fractions (according to manufacturer's instruction). We also collected flow-through, wash (water) and an additional 100% acetonitrile (ACN) elution fraction (total: 11 fractions). Fractions were dried in a centrifugal vacuum concentrator at 45˚C and then stored at -20˚C. Before LC-MS analysis, peptides were re-suspended in 30 µL of 0.1% formic acid (FA) in water.

Ultra/high liquid performance chromatography (U/HPLC). Reversed-phase chromatographic separation utilized Accela Autosampler (Thermo), Accela 600 pump (Thermo) and Acclaim C30 column: 3 µm, 2.1x150 mm (Thermo). The column temperature and tray temperature were kept at 30˚C and 20˚C respectively. Solvent A was composed of 10 mM ammonium acetate in 60:40 methanol:water with 0.2% FA. Solvent B was composed of 10 mM ammonium acetate with 60:40 methanol: chloroform with 0.2% FA. The flow rate was 260 µl/min, and the injection volume was 10 µl. The gradient was 35-100% solvent B over 13.0 min, 100% solvent B over 13.0-13.8 min, 100-35% solvent B over 13.8-14.5 min, 35% solvent B over 14.5-18.0 min, 0% solvent B over 18.0-20.0 min. EASY-nLC 1000 system (Thermo) and Acclaim PepMap RSLC 75 µm × 15 cm, nanoViper column (Thermo) was used for chromatography. Solvent A was composed of 0.1% FA in water. Solvent B was composed of 0.1% FA in ACN (LC-MS grade). The flow rate was 260 µl/min and injection volume was 10 µl. The gradient was 2-5% solvent B over 5.0 min, 5-60% solvent B over 5-70 min, 60-98% solvent B over 70-98 min, 98% solvent B over 98-118 min, 98-2% solvent B over 118-120 min. All solvents used were LC-MS grade unless stated otherwise.

Mass spectrometry. The Q Exactive (Thermo) mass spectrometer was operated under heated electrospray ionization (HESI) in positive and negative mode separately. The spray voltage was 4.4 kV, the heated capillary was held at 310˚C (negative mode) or 350˚C (positive mode) and heater at 275˚C (positive mode). The S-lens radio frequency (RF) level was 70. The sheath gas flow rate was 30 (negative mode) or 45 units (positive mode), and auxiliary gas was 14 (negative mode) or 15 units (positive mode). Full scan used resolution 70,000 with automatic

gain control (AGC) target of 1×10^6 ions and maximum ion injection time (IT) of 100 ms. Data-dependent MS/MS (top10) were acquired using the following parameters: resolution 17,500; AGC 1×10^5; maximum IT 75 ms; 1.3 m/z isolation window; underfill ratio 0.1%; intensity threshold 1×10^3; dynamic exclusion time 3 s. Normalized collision energy (NCE) settings were: 15, 30, 45, 60, 75, 90 (in positive and negative mode separately; total 12 runs per sample).

Lipid identification, quantification, and Processing. Lipid identification and relative quantification were performed using LipidSearch 4.1 software (Thermo). The search criteria for product search were as follows; parent m/z tolerance 5 ppm; product m/z tolerance 5 ppm; quantification: m/z tolerance 5 ppm, retention time tolerance 1 min. The following adducts were allowed in positive mode: +H, $+NH_4$, $+H\text{-}H_2O$, $+H\text{-}2H_2O$, +2H, +Na, +K and negative mode: -H, +HCOO, $+CH_3COO$, -2H, -Cl. All classes using LipidSearch nomenclature was selected and searched. Positive and negative mode identifications at different NCE were aligned allowing calculation of unassigned peaks with following settings: product search; alignment method max; retention time tolerance 0.1 min; filters: top rank, main isomer peak; M-score 0; molecular lipid identification grade: A-D.

Protein identification and quantification. The acquired raw files were analyzed with Proteome Discoverer 2.2 (Thermo) using the SEQUEST HT engine. The data was searched against Mus musculus entries (Swiss-Prot + TrEMBL, UniProt 8/14/2018). Search parameters included: precursor mass tolerance 10 ppm and 0.02 Da for fragments, 2 missed trypsin cleavages, oxidation (Met) and acetylation (protein N-term) as variable modifications, carbamidomethylation (Cys) as a static modification. Percolator PSM validation used the following parameters: strict FDR of 0.01, relaxed FDR of 0.1, maximum ΔCn of 0.05, validation based on q-value. For label-free quantification (LFQ), the Minora Feature Detector was used along with the Feature Mapper and Precursor Ions Quantifier. Data were filtered by peptide filter: medium (FDR 0.1) and high confidence (FDR 0.01) and protein filter: medium (FDR 0.1) and high confidence (FDR 0.01).

Optic Nerve Regeneration Profiling. Lipid profiling was performed from the retina and ON samples during Wnt3a and Zymosan-induced retinal ganglion cells regeneration through extractive mass spectrometry-based lipidomics. The experimental groups were: intact control (control), optic nerve (ON) crush + vehicle (crush) and ON crush + Zymosan + CPT-cAMP (regeneration). Zymosan is a yeast cell wall preparation traditionally used to induce sterile inflammation experimentally. The addition of a cell-permeable cAMP analog (CPT-cAMP) potentiates Zymosan’s action but cannot induce ON regeneration when administered alone (Kurimoto et al., 2010). The ONs were collected 3, 7, 14 and the retinas 7, 14 days post-crush. Zymosan + CPT-cAMP treatment potently increased the amount of axon regeneration. Time points were chosen based on previous published reports. After axotomy, most RGCs die within 2 weeks (Kermer et al., 2001). The intravitreal inflammatory response presents a hazy vitreous on day 3 post-crush and concomitant Zymosan injection (Stark et al., 2018). On day 7, the ON crush site is densely occupied by Iba1 positive macrophages/microglia (Stark et al., 2018). Zymosan + CPT-cAMP doubles the number of live RGCs in retina 2 weeks after ON crush (Kurimoto et al., 2010). After a chloroform-methanol based extraction, lipid samples were analyzed using high-performance liquid chromatography (HPLC) with C30 column coupled to a Q Exactive mass spectrometer operated in a data-dependent mode. Peak identification as well as relative quantification were performed in LipidSearch software. Lists of species and their

relative abundances were uploaded to MetaboAnalyst (Xia and Wishart, 2011) for statistical analysis.

Bioinformatics and Tool Development. Growth cone proteomic and lipidomic datasets were normalized and validated through a data transformation pipeline with three stages prior to statistical and bioinformatic analysis. The first stage took the raw data captured as input and removed incomplete data instances. The second filtered data by mean intensity value. The third stage transformed (log_2) and normalized (quantile) data (Figure S2 A-E) (Lee et al., 2012; (Mertens, 2016). For cell-type specific enrichment analysis we used R package pSI in R and CSEA data package and tool for specific and enriched transcripts with published bacTRAP cell types as background. (Dougherty et al., 2010; (Heintz, 2004; (Xu et al., 2014) P-values were estimated using either a BH or FDR adjusted comparison test with significances quoted at $p<0.05$, 0.01, or 0.001. Growth cone proteomic and lipidomic comparisons across developmental stages and fraction were compared using either two-way t-tests, ANOVA, or Pearson correlations with P-values presented as permutation-based FDR-adjusted with significance quoted at $p<0.05$. Correlation analyses were performed using MetaboAnalyst 4.0, STATA 14.2, or R. (Chong et al., 2018) Unsupervised principle component analyses were performed using the R package ggplot2 and MetaboAnalyst 4.0 with SVD with imputation method and Pareto scaling of rows. (Wickham, 2009) Heat maps were made using the R package phetmap and MetaboAnalyst 4.0 utilizing a Euclidean distance metric and Ward clustering algorithm. Unsupervised hierarchical clustering was performed separately for GCM and GCP fractions, as well as for the growth cone lipidome and proteome, with the similarity of each developmental stage assessed by a Euclidean distance metric and Ward clustering algorithm. For the categorization of GCM lipidome, we utilized a k-means clustering algorithm with a 2-component cluster solution. To identify important protein and lipid patterns along development, we performed a Pearson correlation for the determination of molecules that increase or decrease linearly in abundance along the five developmental stages using a $p<0.05$ cutoff. To identify protein signatures that predict different developmental stages, we performed a PLS regression and combined variable importance in projection scores with coefficients from Pearson correlation. Thus, this analysis provided information on proteins that change linearly along development and are predictive of the different developmental stages. Protein-protein:lipid correlations in Figure 3B were done in four steps. Firstly, STRING protein-protein interactions were identified in Cytoscape, with integrated data with CORUM database for protein complex information. (Giurgiu et al., 2019) Second, identified interacting proteins were determined for putative lipid binding or lipid-related metabolic functions through MetScape and LipidMaps database (Karnovsky et al., 2012; (O'Donnell et al., 2019). Third, each individual protein in an interaction network was correlated with lipid classes in early and late development. Fourth, we identified proteins that had similar correlation patterns (i.e., were either significantly ($p<0.05$) *positively* or *negatively* correlated with a lipid class) and then averaged their correlation coefficients by transforming coefficients to Fisher's Z values, averaging these values, and back transforming to Pearson coefficients. KEGG, Reactome, and GO-based analyses (MF, CC, and BP) were performed using the Database for Annotation, Visualization and Integrated Discovery (DAVID) and ClueGO with semantic clustering and similarity measures performed using REViGO and NaviGO. (Ding et al., 2018; (Huang et al., 2007; (Mlecnik et al., 2019; (Supek et al., 2011) Lipid ontologies were performed using LION for biophysical and chemical properties and the R package Rodin for category, class, and subclass visualization and enrichment. The ON regeneration lipidomic datasets (Wnt3a and Zymosan) were normalized individually followed

by ComBat batch correction to mitigate signal drift and technical heterogeneity. To compare the predictive ability of lipid species for predicting regeneration, ROC analysis was used and AUCs were calculated. In addition, we have created a software tool (GC-Insights) to integrate multi-omic data sets from isolated neuronal growth cones.

GC-Insights is a web-based application to enable researchers to compare user-defined genes and lipid class sets for relative abundance of proteins and lipids at various developmental stages. It provides an interactive query mechanism, visualizations and analysis, experiment reports and data capture. GC-Insights is written in Java and Python and is deployed in Heroku, a cloud platform that supports Java, Node.js, Scala, Clojure, Python, PHP, and Go programming languages. The authors considered and created a prototype using R Shiny package and the shinyapps.io platform from RStudio. The Heroku platform was selected based on the robust programming languages support and the scalability of the platform. We envision extending this application to work with additional datasets. The power of Java combined with the availability of vast Python packages provides scalability and extensibility. GC-Insights uses Machine Learning techniques to analyze, visualize, and cluster the data. The user interface is a web-based GUI interface providing the user the ability to interactively perform queries on the datasets, view visualizations and analysis results. The user selects the omics layer, gene name or lipid class, fraction, and one or more developmental stages of interest. The application performs the analysis and outputs visualizations and results. The output depends on the number of inputs and the comparative groups selected. In addition, the user is able to download an experiment report in PDF format and download the data used in the query in CSV format. There are four datasets. Each dataset contains either protein or lipid concentration values. The values are molecular abundance, as determined by the area under the curve of the m/z ratio expressed in micro scale weight units. Within each fraction (GCM or GCP) there are concentration values across five developmental age groups There are multiple replicates of each group to control for experimental error. The datasets were normalized and validated through a data transformation pipeline with four stages. The first stage took the raw data captured as input and removed incomplete data instances. The second replaced UniProt protein IDs with gene names. The third sorted the data instances by gene name and lipid class. The fourth and final stage applied a log2 transformation and created the normalized dataset.

Data availability and Accessions

All proteomics data associated with the paper is being deposited in PRIDE proteomeXchange (http://www.proteomexchange.org/), (accession IDs PXD012134 and PXD016197). Transcriptomic expression data used are deposited at the Gene Expression Omnibus (GEO) (accession numbers: GSE32309, GSE360068; GSE38668; GSE30626; GSE13379; GSE43164, GSE13344 and GSE25219, SRP031888). All lipidomics data will be available at the Metabolomics Workbench, http://www.metabolomicsworkbench.org, (Project IDs: PR000856). Processed growth cone proteomic and lipidomic data can be accessed at the following open access web utility: https://gcinsights.herokuapp.com/. Any additional data may be made available upon reasonable request to the corresponding author.

References

Chong, J., Soufan, O., Li, C., Caraus, I., Li, S., Bourque, G., Wishart, D.S., and Xia, J. (2018). MetaboAnalyst 4.0: towards more transparent and integrative metabolomics analysis. Nucleic Acids Res *46*, W486-W494.

Ding, Z., Wei, Q., and Kihara, D. (2018). Computing and Visualizing Gene Function Similarity and Coherence with NaviGO. Methods Mol Biol *1807*, 113-130.

Dougherty, J.D., Schmidt, E.F., Nakajima, M., and Heintz, N. (2010). Analytical approaches to RNA profiling data for the identification of genes enriched in specific cells. Nucleic Acids Res *38*, 4218-4230.

Ellis, L., Wallis, I., Abreu, E., and Pfenninger, K.H. (1985). Nerve growth cones isolated from fetal rat brain. IV. Preparation of a membrane subfraction and identification of a membrane glycoprotein expressed on sprouting neurons. J Cell Biol *101*, 1977-1989.

Estrada-Bernal, A., Sanford, S.D., Sosa, L.J., Simon, G.C., Hansen, K.C., and Pfenninger, K.H. (2012). Functional complexity of the axonal growth cone: a proteomic analysis. PloS one *7*, e31858.

Giurgiu, M., Reinhard, J., Brauner, B., Dunger-Kaltenbach, I., Fobo, G., Frishman, G., Montrone, C., and Ruepp, A. (2019). CORUM: the comprehensive resource of mammalian protein complexes-2019. Nucleic Acids Res *47*, D559-D563.

Heintz, N. (2004). Gene expression nervous system atlas (GENSAT). Nat Neurosci *7*, 483.

Huang, D.W., Sherman, B.T., Tan, Q., Collins, J.R., Alvord, W.G., Roayaei, J., Stephens, R., Baseler, M.W., Lane, H.C., and Lempicki, R.A. (2007). The DAVID Gene Functional Classification Tool: a novel biological module-centric algorithm to functionally analyze large gene lists. Genome Biol *8*, R183.

Karnovsky, A., Weymouth, T., Hull, T., Tarcea, V.G., Scardoni, G., Laudanna, C., Sartor, M.A., Stringer, K.A., Jagadish, H.V., Burant, C.*, et al.* (2012). Metscape 2 bioinformatics tool for the analysis and visualization of metabolomics and gene expression data. Bioinformatics *28*, 373-380.

Kermer, P., Klocker, N., Weishaupt, J.H., and Bahr, M. (2001). Transection of the optic nerve in rats: studying neuronal death and survival in vivo. Brain research Brain research protocols *7*, 255-260.

Kurimoto, T., Yin, Y., Omura, K., Gilbert, H.-y., Kim, D., Cen, L.-P., Moko, L., Kugler, S., and Benowitz, L.I. (2010). Long-distance axon regeneration in the mature optic nerve: Contributions of Oncomodulin, cAMP, and pten gene deletion. The Journal of neuroscience : the official journal of the Society for Neuroscience *30*, 15654-15663.

Lee, J., Park, J., Lim, M.S., Seong, S.J., Seo, J.J., Park, S.M., Lee, H.W., and Yoon, Y.R. (2012). Quantile normalization approach for liquid chromatography-mass spectrometry-based metabolomic data from healthy human volunteers. Anal Sci *28*, 801-805.

Mertens, B.J.A. (2016). Transformation, Normalization, and Batch Effect in the Analysis of Mass Spectrometry Data for Omics Studies (Switzerland: Springer International Publishing).

Mlecnik, B., Galon, J., and Bindea, G. (2019). Automated exploration of Gene Ontology term and pathway networks with ClueGO-REST. Bioinformatics.

O'Donnell, V.B., Dennis, E.A., Wakelam, M.J.O., and Subramaniam, S. (2019). LIPID MAPS: Serving the next generation of lipid researchers with tools, resources, data, and training. Sci Signal *12*.

Park, K.K., Liu, K., Hu, Y., Kanter, J.L., and He, Z. (2010). PTEN/mTOR and axon regeneration. Exp Neurol *223*, 45-50.

Park, K.K., Liu, K., Hu, Y., Smith, P.D., Wang, C., Cai, B., Xu, B., Connolly, L., Kramvis, I., Sahin, M.*, et al.* (2008). Promoting axon regeneration in the adult CNS by modulation of the PTEN/mTOR pathway. Science *322*, 963-966.

Patel, A.K., Park, K.K., and Hackam, A.S. (2017). Wnt signaling promotes axonal regeneration following optic nerve injury in the mouse. Neuroscience *343*, 372-383.

Pfenninger, K.H., Ellis, L., Johnson, M.P., Friedman, L.B., and Somlo, S. (1983). Nerve growth cones isolated from fetal rat brain: subcellular fractionation and characterization. Cell *35*, 573-584.

Stark, D.T., Anderson, D.M.G., Kwong, J.M.K., Patterson, N.H., Schey, K.L., Caprioli, R.M., and Caprioli, J. (2018). Optic Nerve Regeneration After Crush Remodels the Injury Site: Molecular Insights From Imaging Mass Spectrometry. Invest Ophthalmol Vis Sci *59*, 212-222.

Supek, F., Bosnjak, M., Skunca, N., and Smuc, T. (2011). REVIGO summarizes and visualizes long lists of gene ontology terms. PloS one *6*, e21800.

Trzeciecka, A., Stark, D.T., Kwong, J.M.K., Piqueras, M., Bhattacharya, S.K., and Caprioli, J. (2019). Comparative lipid profiling dataset of the inflammation-induced optic nerve regeneration. Data Brief *24*, 103950.

Wickham, H. (2009). Ggplot2 : elegant graphics for data analysis (New York: Springer).

Xia, J., and Wishart, D.S. (2011). Web-based inference of biological patterns, functions and pathways from metabolomic data using MetaboAnalyst. Nature protocols *6*, 743-760.

Xu, X., Wells, A.B., O'Brien, D.R., Nehorai, A., and Dougherty, J.D. (2014). Cell type-specific expression analysis to identify putative cellular mechanisms for neurogenetic disorders. J Neurosci *34*, 1420-1431.

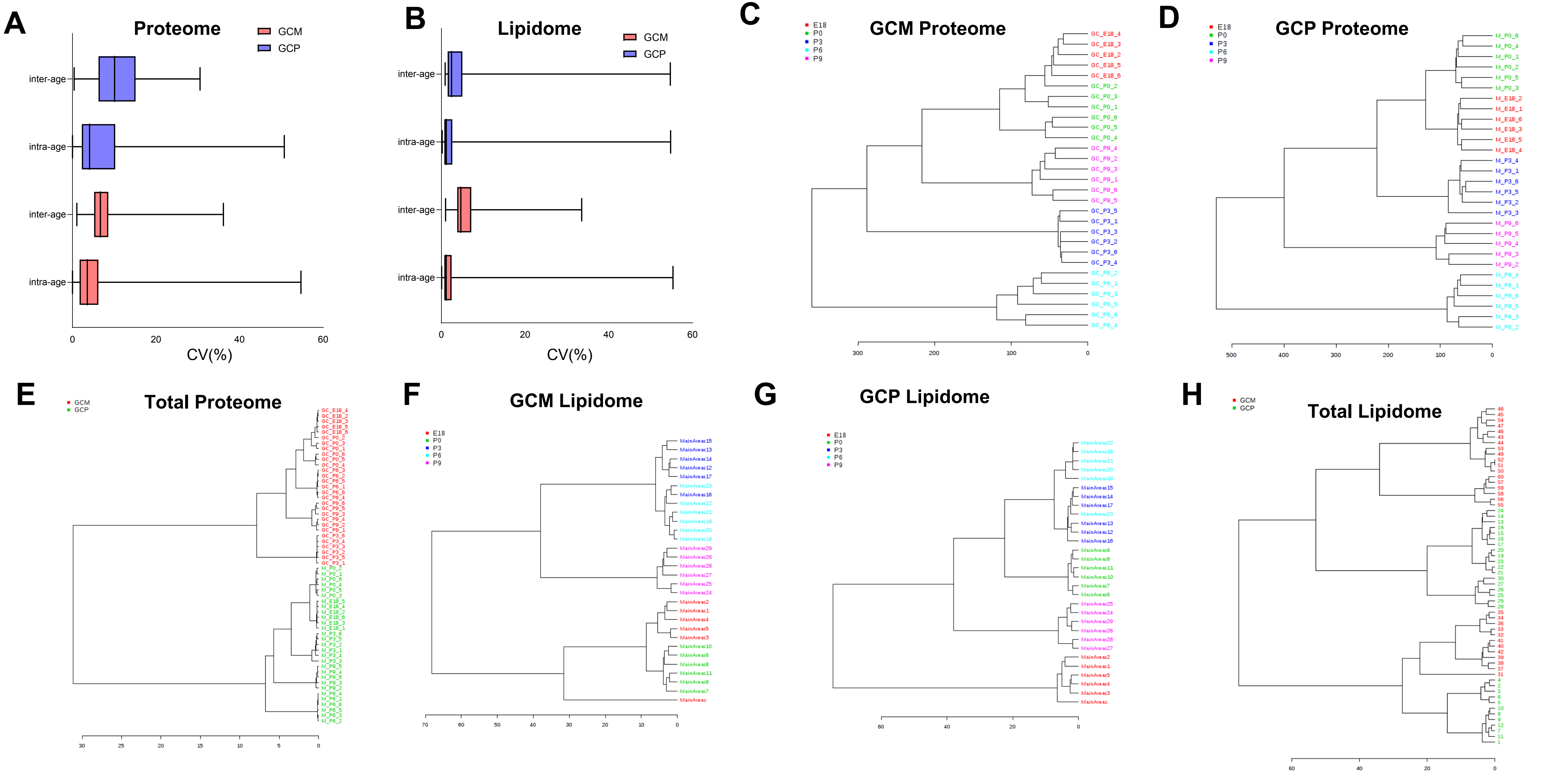

Figure S1, related to Figure 1. Examination of growth cone proteomic and lipidomic reproducibility. (**A-B**) Coefficient of variation analysis of proteomic (A) and lipidomic (B) data in the growth cone membrane (GCM) and growth cone particulate (GCP) fractions. (C-H) Unsupervised hierarchical clustering of growth cone proteomic (**C-E**) and lipidomic (**F-H**) data across developmental stages and between fractions utilizing a Euclidean distance measure and a Ward clustering algorithm. Total proteomics data clustered 100% of GCM and GCP samples by fraction (E) and into their respective developmental stages (C, D). Total lipidomic of GCM and GCP samples showed significant overlap in clustering analysis (H), while 93% and 96% of the GCM and GCP samples respectively, clustered into their age groups (F, G).

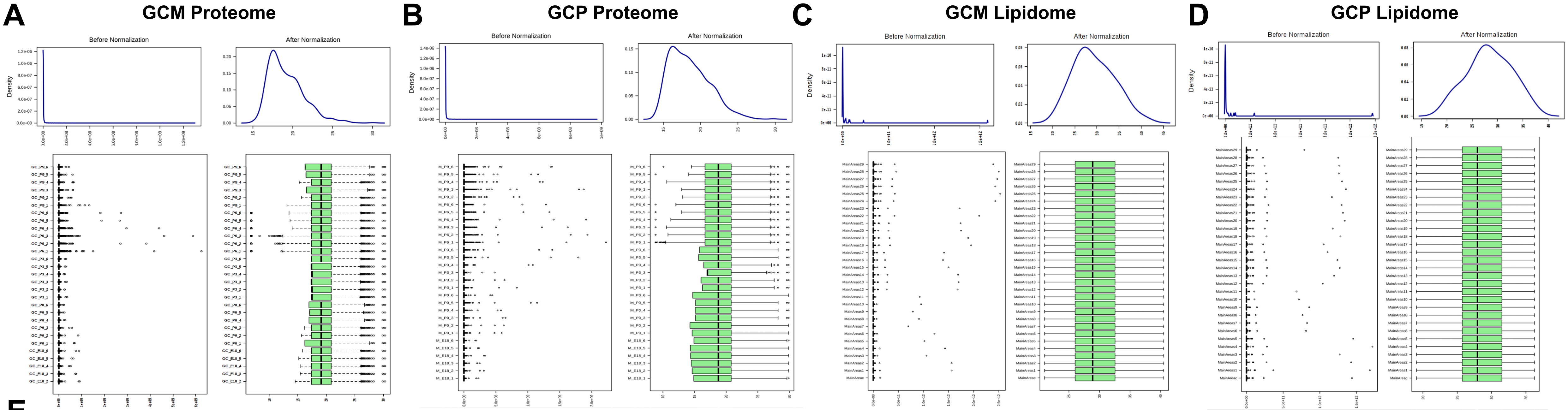

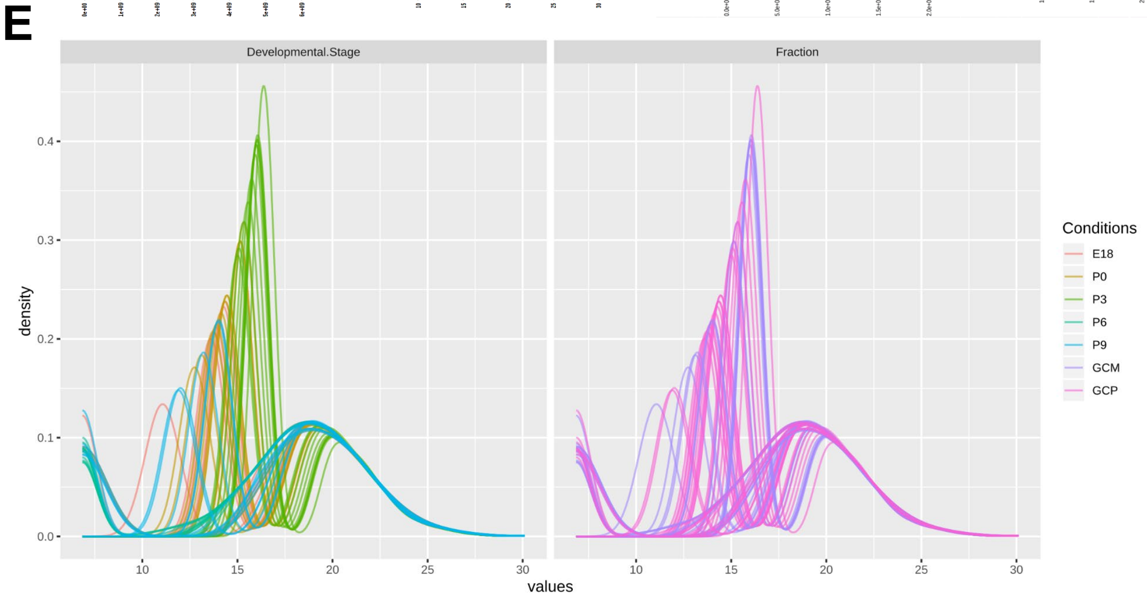

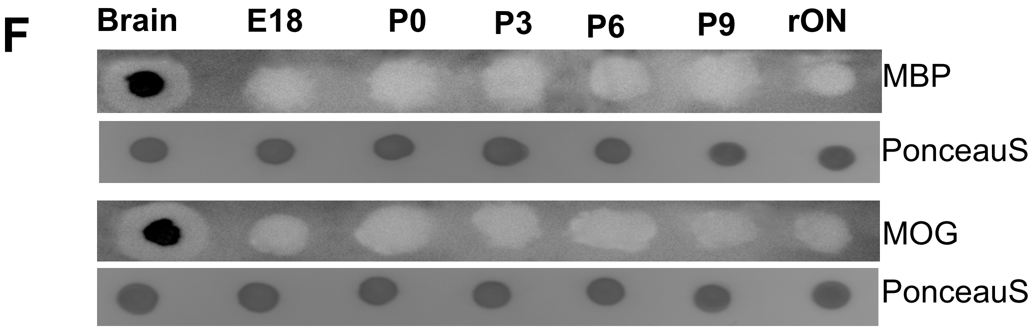

Validation of fractions using mass spectrometry*

	Brain	E18	P0	P3	P6	P9	rON
MBP	√	X	X	X	X	X	X
MOG	√	X	X	X	X	X	X
Ncam1	√	√	√	√	√	√	√
GAP43	√	√	√	√	√	√	√

Figure S2, related to Figure 1. Data normalization and validation. (**A-D**) Growth cone membrane (GCM) and growth cone particulate (GCP) proteomic and lipidomic peak area values were filtered by mean intensity value, quantile normalized, and log2 transformed to mitigate technical variation and create a normal distribution for statistical analyses. Visualization of the density plot (top) before and after normalization shows a more normal distribution. A box plot of the value distribution across samples (bottom) shows that samples are suitable for comparison. (**E**) Combined density plot of data across developmental stage and fraction after normalization. (**F**) validation of fractions using dot blot (0.5 µg load) using antibodies to myelin basic protein (MBP) and myelin oligodendrocyte protein (MOG) as indicated. Brain extract (positive control), regenerating optic nerve (rON) and GC fractions (E18-P9) as indicated. PonceauS staining prior to probing with antibody to demonstrate protein load as indicated. Bottom panel data using mass spectrometry. *Based on capture of at least 4 peptides (≥22 sequence coverage). HEK293 cell extract was used as a negative control for these dot blots (not shown).

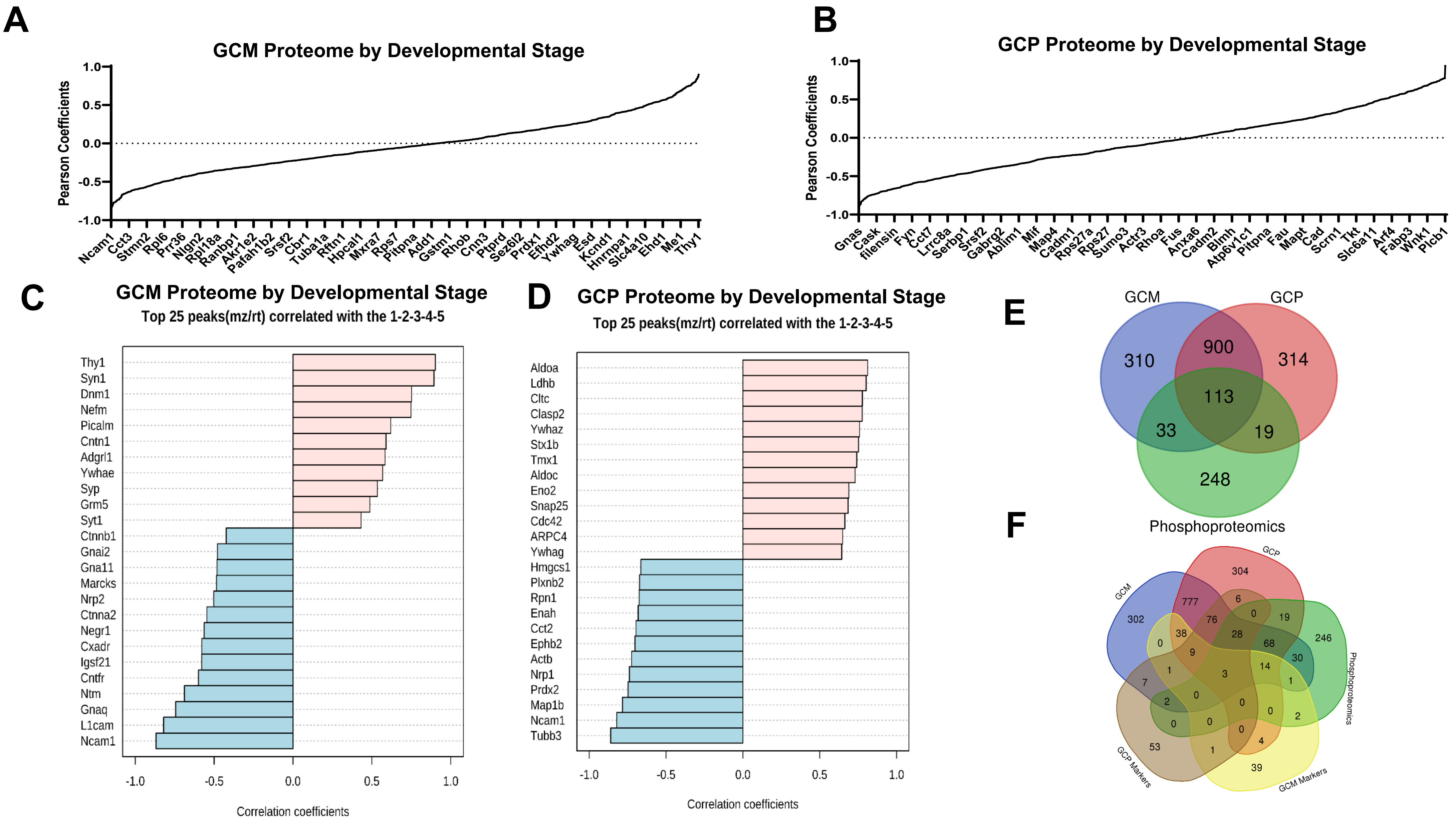

Figure S3, related to Figures 1 and 3. GCM and GCP proteome correlations with developmental stage. (A, B) Growth cone membrane (GCM) (**A**) and growth cone particulate (GCP) (**B**) proteomic correlations. Only a subset of proteins are labeled. Y-axis are Pearson coefficients (R^2) with negative values indicating proteins whose abundances decrease as age increases and positive correlations indicating proteins whose abundance increase as age increases (E18 -> P9). (C, D) Correlation coefficients for GCM Proteome (**C**) and GCP proteome (**D**) by developmental stage. Upregulated and downregulated proteins have been shown in red and blue respectively. (**E, F**) Comparison of recent identified proteome as part of GC phosphoproteomics (Kawasaki et al., 2018) with our GCM and GCP proteome.

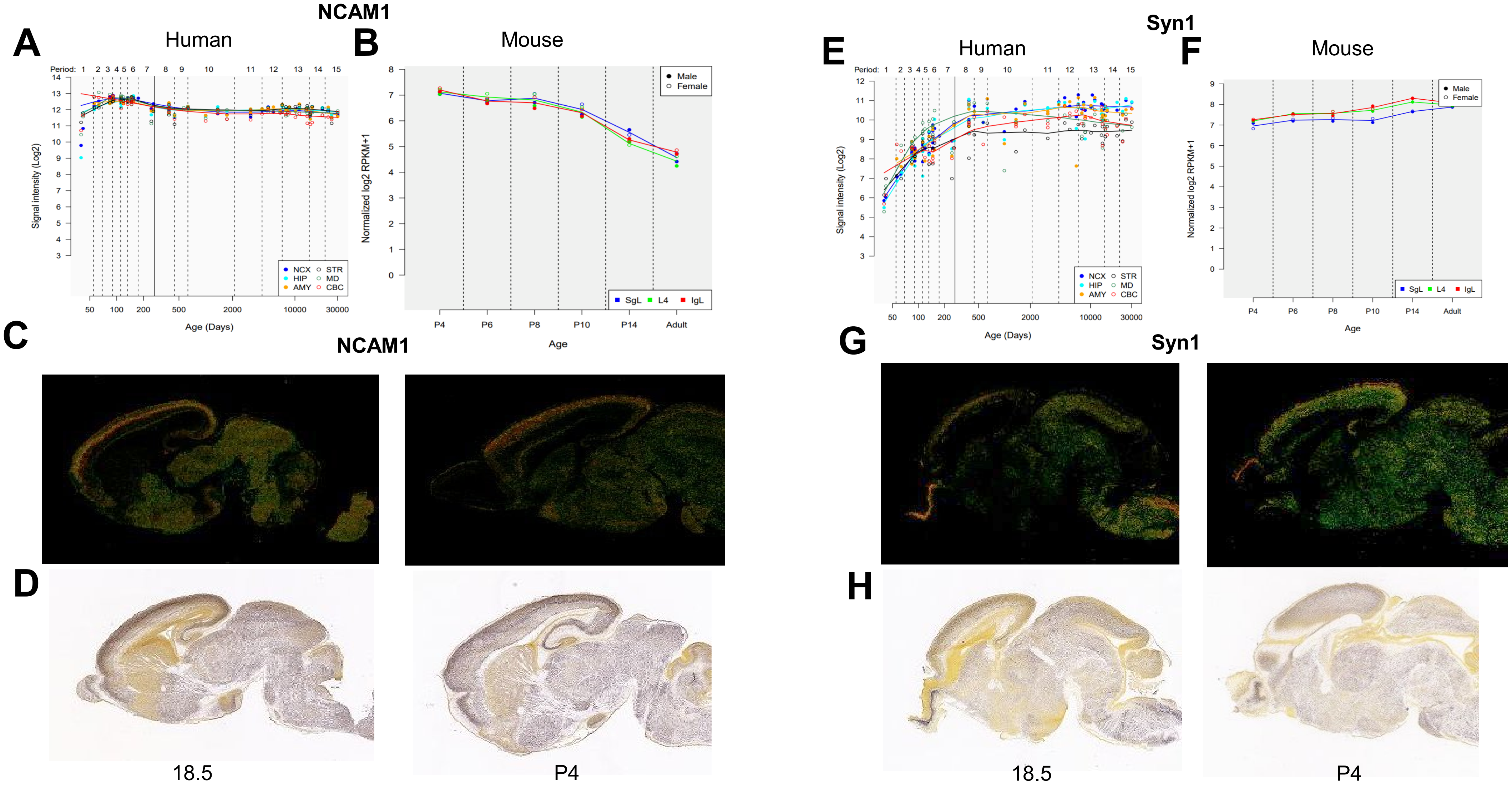

Figure S4, related to Figures 1 and 3. Validation of proteomic correlation analyses utilizing transcriptomic data. (**A, E**) Transcriptome data from two datasets (GSE13344 and GSE25219) for the developing and adult human brain for NCAM1 and Syn1. Data consists of over 1,340 tissue samples from both hemispheres of postmortem human brains. Specimens range from embryonic development to adulthood and are representative of both males and females from multiple ethnicities. A total of 16 brain regions were sampled: the cerebellar cortex (CBC) , mediodorsal nucleus of the thalamus (MD), striatum, amygdala (STR), hippocampus (HP), and 11 areas of the neocortex. (**B, F**) Transcriptome data generated using Illumina high throughout sequencing of over 36 samples at infragranular layers (IgL), granular layer (L4), and supragranular layers (SgL) from the primary somatosensory cortex of mouse (C57BL/6J) brain at postnatal day (P) P4, P6, P8, P10, P14 and P180 (adult) for NCAM1 and Syn1 (SRP031888). (C, D, G, H) Expression (**C, G**) and in situ hybridization with HP yellow counterstain (**D, H**) images from Allen Brain Atlas of NCAM1 and Syn1 in mouse (C57BL/6J) at 18.5 and P4 developmental stages using the ScanScope® automated slide scanner (Aperio Technologies, Inc; Vista, CA) equipped with a 20x objective and Spectrum software.

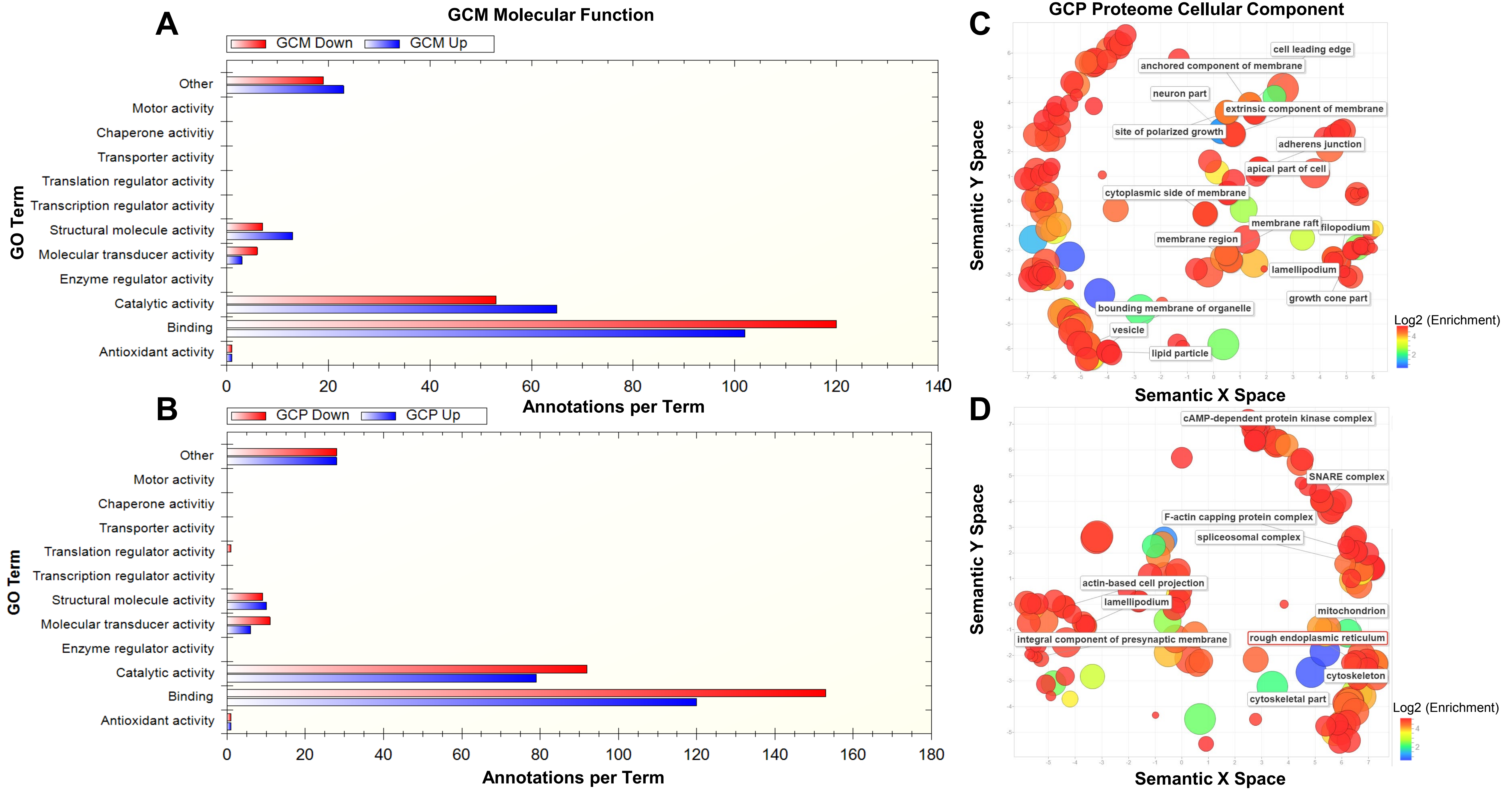

Figure S5, related to Figure 2. GCM and GCP proteome molecular function and cellular component analysis. (**A, B**) GO molecular function analysis of parent terms in the GCM (A) and GCP (B) proteome that linearly decrease with developmental stage (red) or increase with developmental stage (blue). X-axis details the number of annotations that fell within each GO term. (**C, D**) Semantic clustering of GO cellular component terms in the GCM (C) and GCP (D) proteome. Colors represent the enrichment in each term.

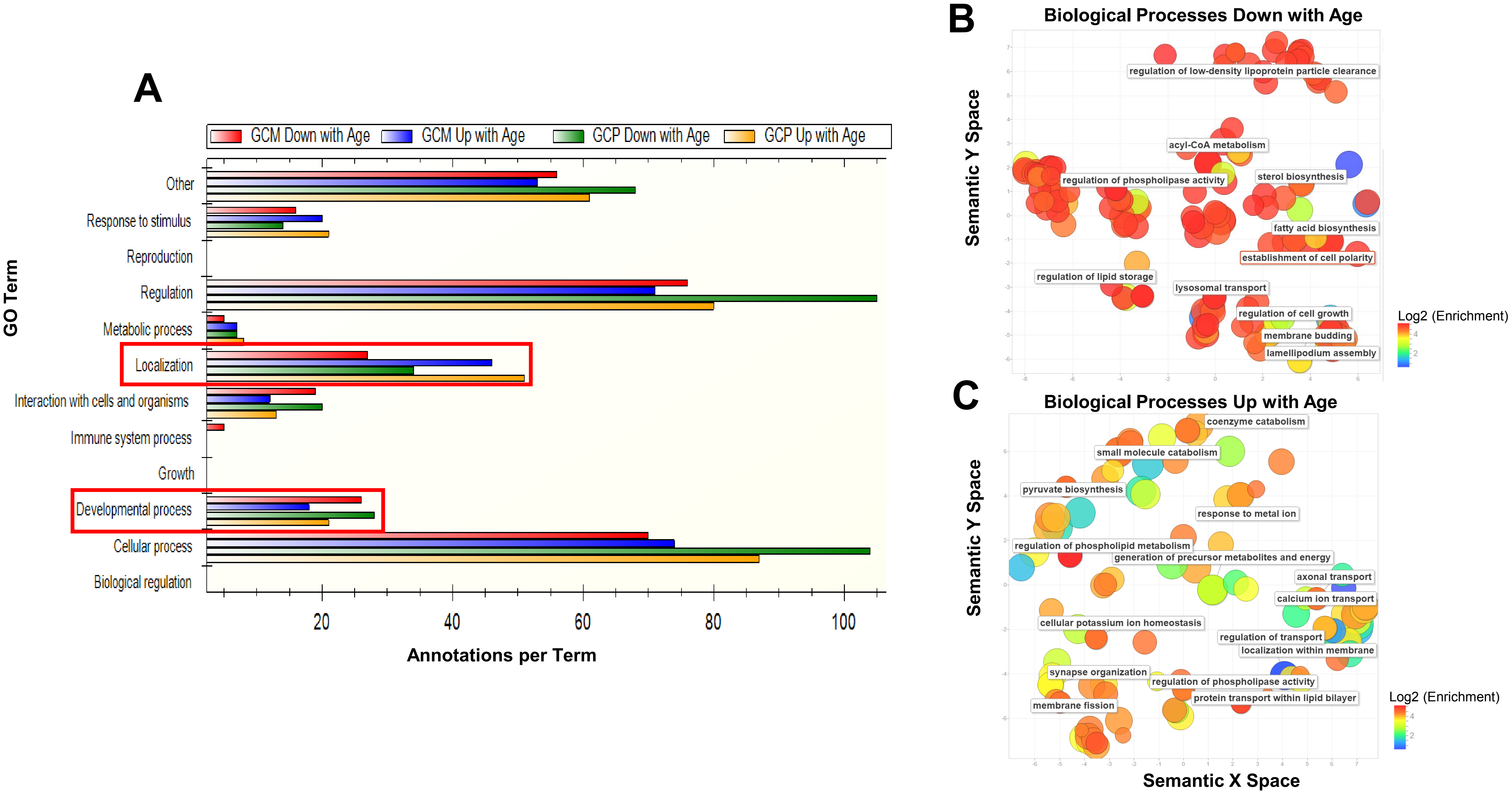

Figure S6, related to Figure 2. Growth cone biological process analysis. (**A**) GO biological processes in the GCM and GCP proteome grouped by fraction and whether there is an increase or decrease in abundance with developmental stage. “Localization” and “developmental process” terms (red rectangular brackets) were processes that revealed commonalties in groups.(B, C) Semantic clustering of biological process terms grouped by decreased with age (**B**) or increases with age (**C**).

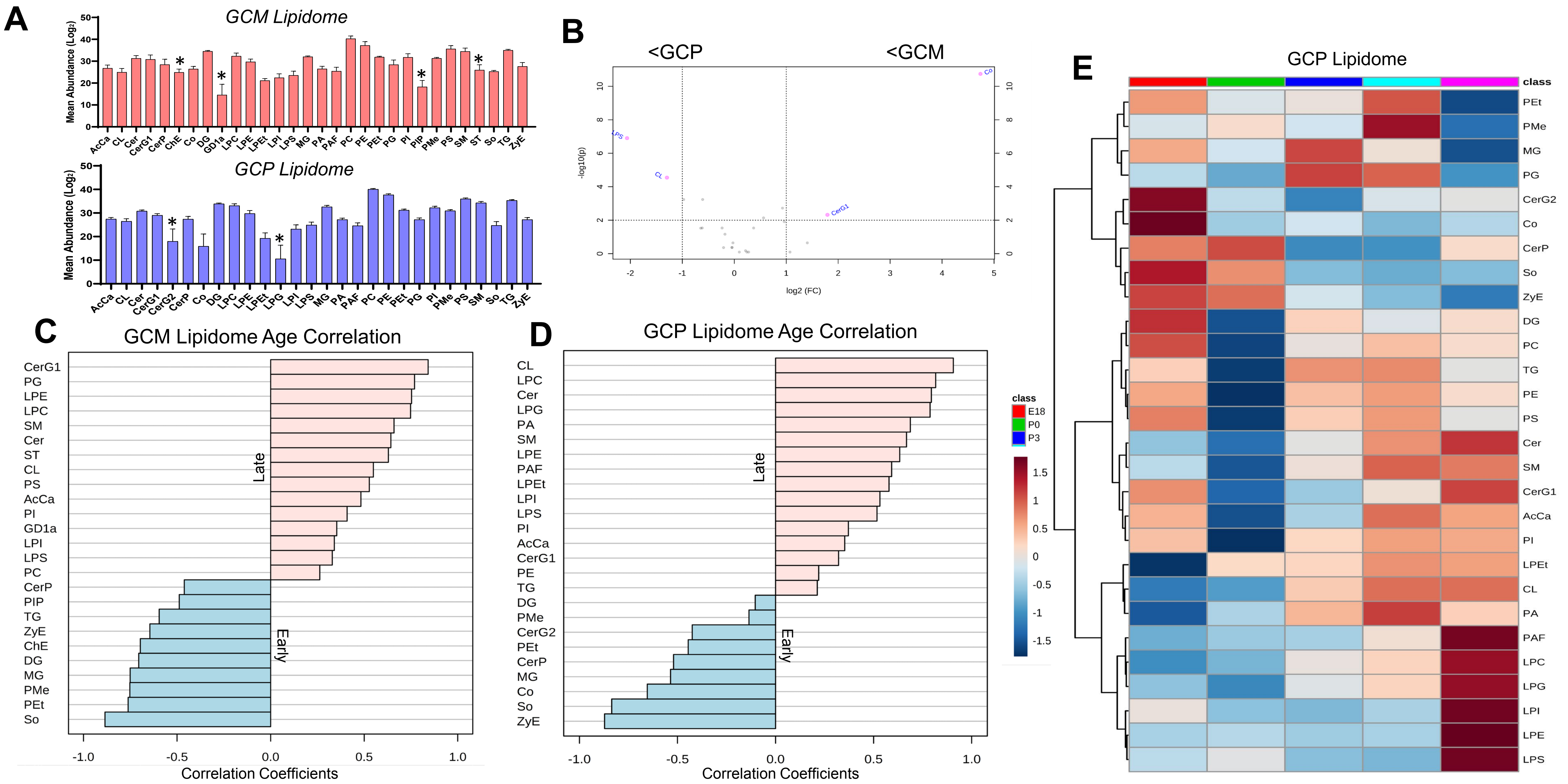

Figure S7, related to Figure 3. Growth cone lipidomic analysis. (**A**) Average abundance of all lipid classes in the GCM (top, red) and GCP (bottom, blue); data are mean ± s.d.; stars (*) indicate unique lipid class to fraction (n=60 mice). (**B**) Volcano plot of common lipid classes that are significantly different between GCM and GCP (FDR adjusted $p < 0.01$; fold-change threshold > 2). Lipid classes significantly greater in the GCP are shown one the left, while classes greater in the GCM are on the right. (C, D) Pearson correlation of lipid classes in the GCM (**C**) and GCP (**D**) that most significantly positively (red, “late”) and negatively (blue, “early) correlated with a linear increase along developmental stage. (**E**) , Heat map of mean abundance of lipid classes across developmental stage in GCP; Euclidian distance measure, Ward clustering algorithm, features auto scaled.

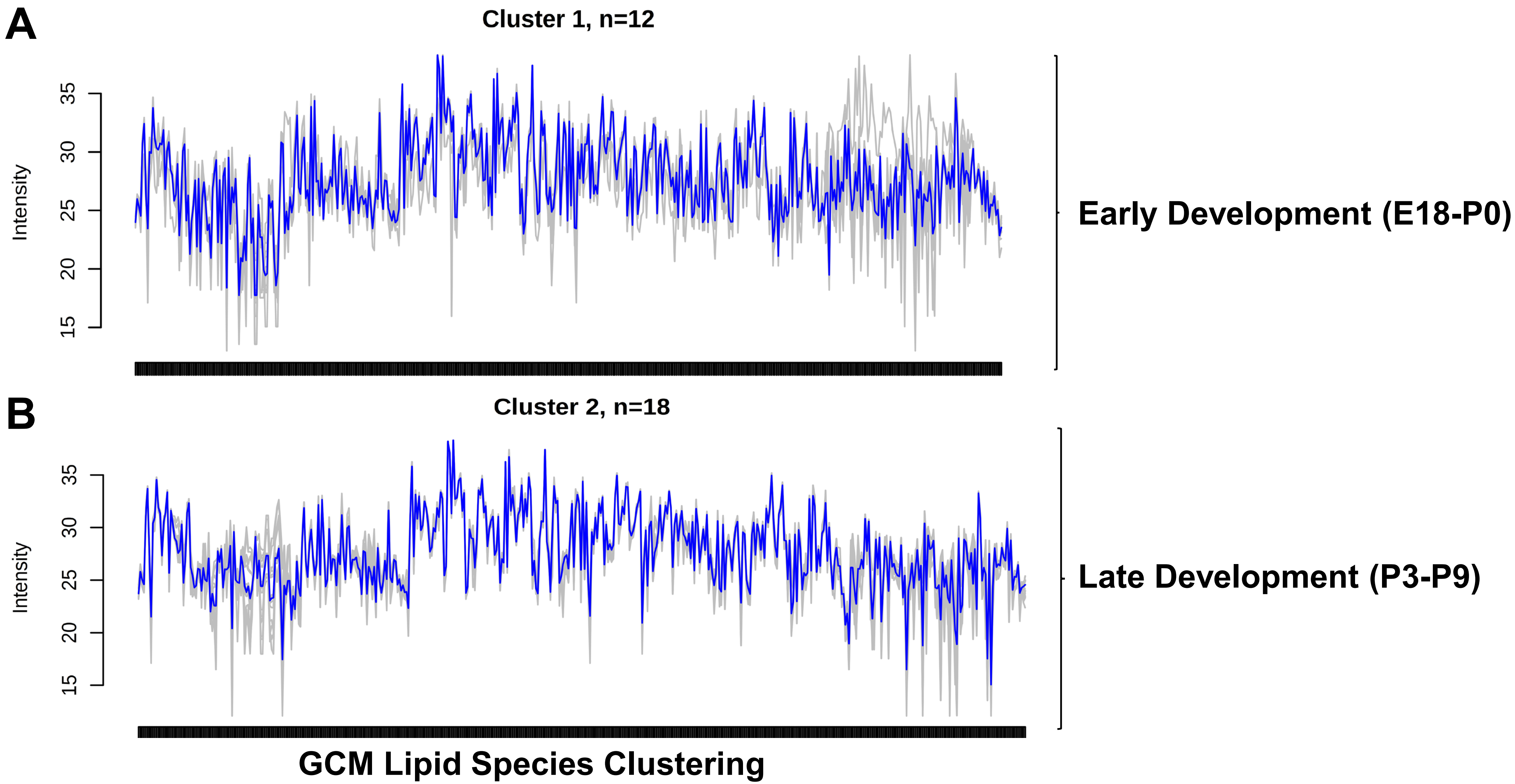

Figure S8, related to Figure 3. K-mean clustering of GCM lipid species. (A, B) Clustering of GCM lipid species utilizing a k-means algorithm with a 2-cluster solution to observe sample grouping. E18-P0 samples (n=12) fully clustered into one group (**A**), while P3-P9 samples (n=18) fully clustered into another group (**B**). We have labeled the E18-P0 group "early development" and the P3-P9 group "late development."

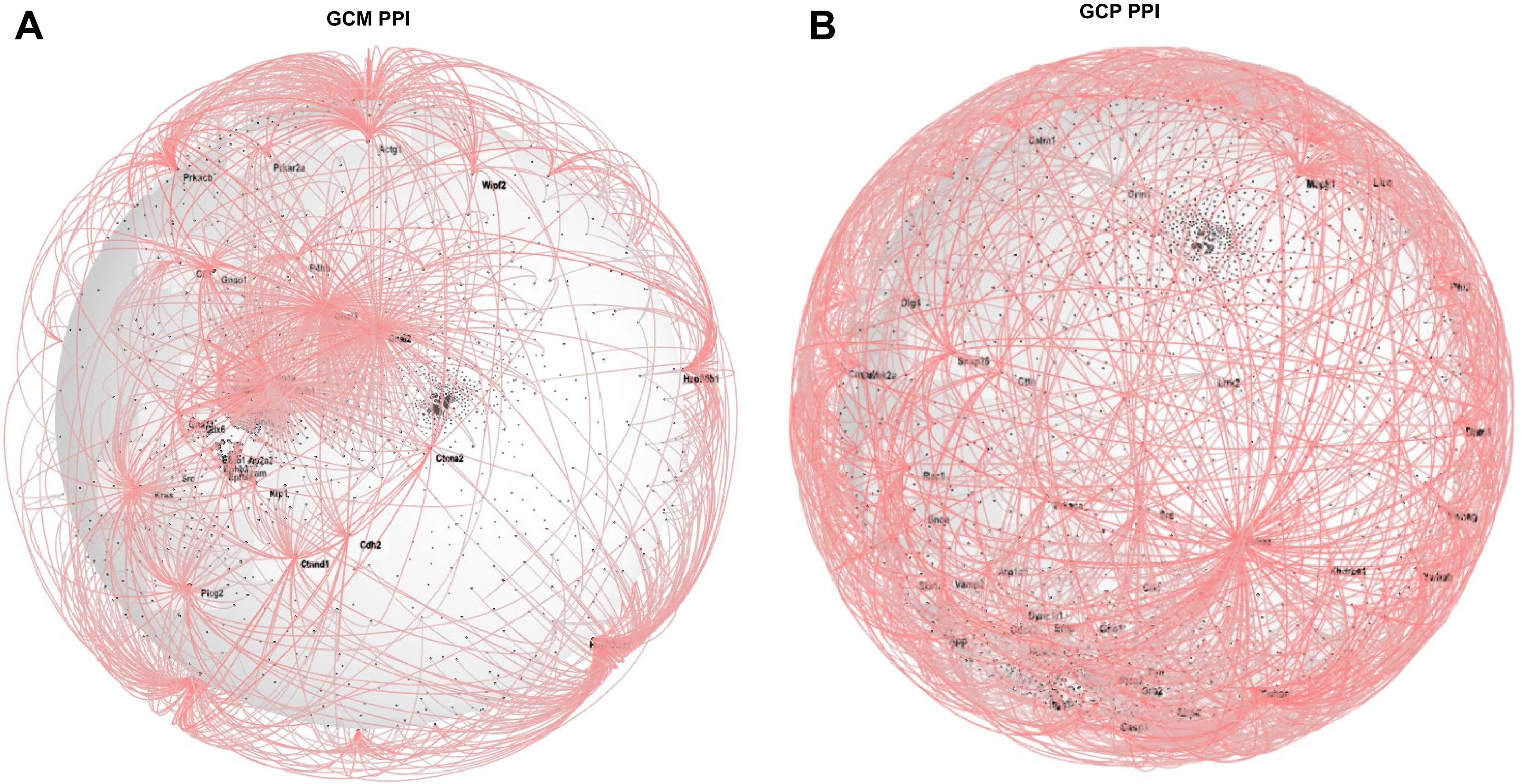

Figure S9, related to Figure 4. Protein-protein interaction and protein complex analysis. (A, B) Global string protein-protein interactions (PPI) in the GCM (**A**) and GCP (**B**) yielded a significant number of interactions in both fractions; red lines indicate protein interactions (confidence score>900).

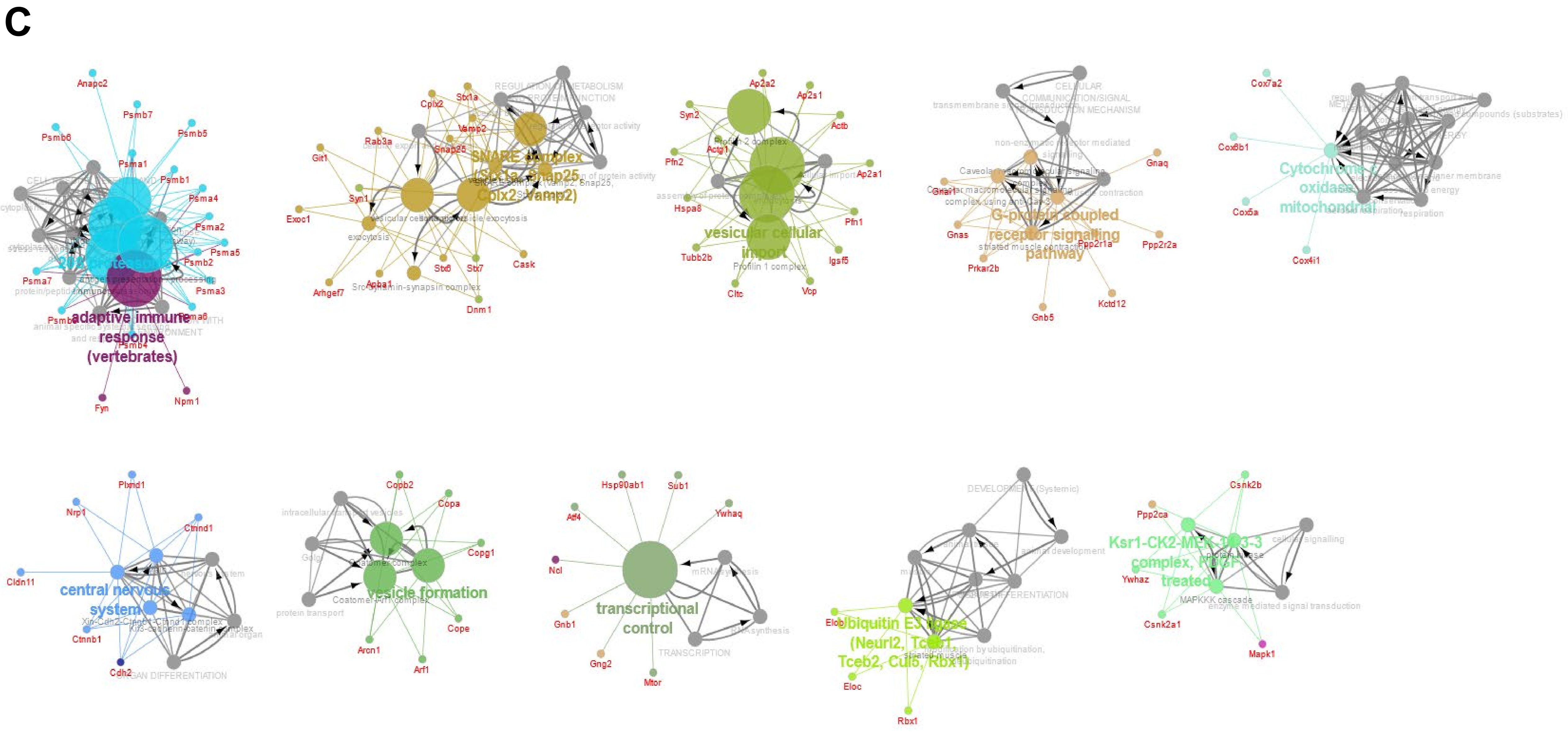

Figure S9, related to Figure 4. Protein-protein interaction and protein complex analysis. (**C**) Selected CORUM protein complexes identified from PPI coupled with gene ontologies for functional comparisons. (part 1). [The network panel figures have been divided into two parts for presenting a proper magnified and legible view. This is part 1 of Figure S9C].

C

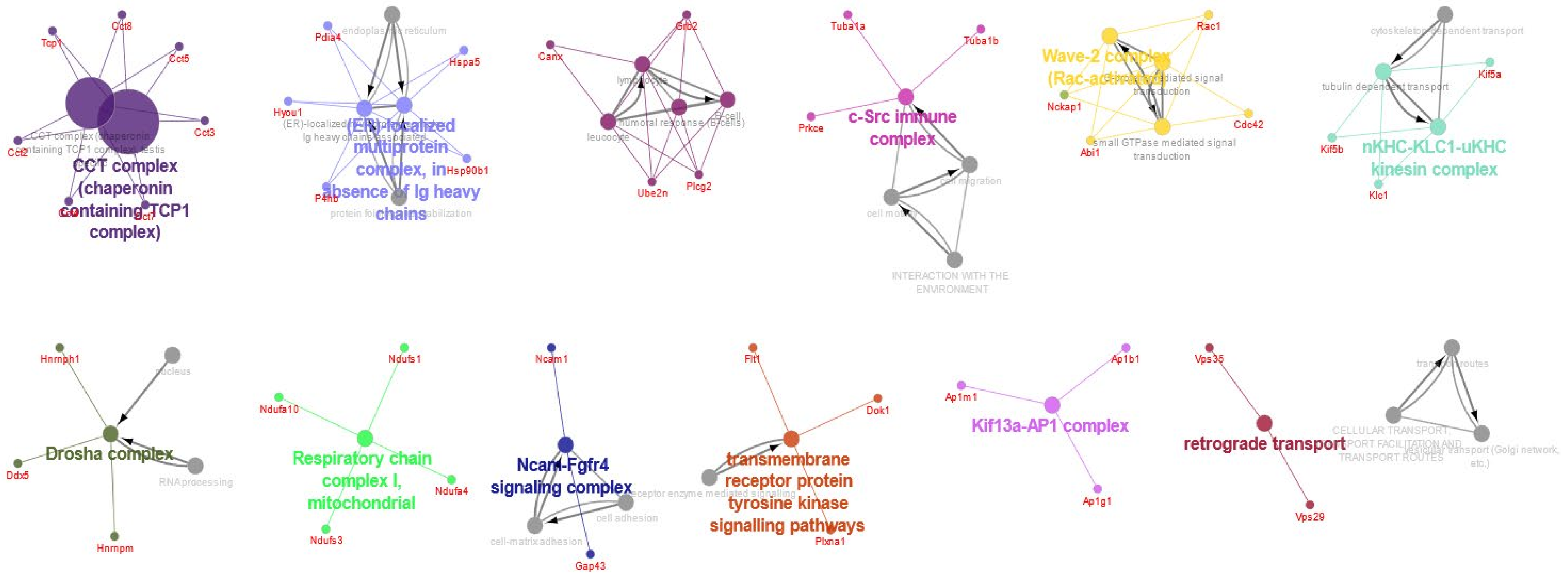

Figure S9, related to Figure 4. Protein-protein interaction and protein complex analysis. (**C**) Selected CORUM protein complexes identified from PPI coupled with gene ontologies for functional comparisons (part 2). [The network panel figures have been divided into two parts for presenting a proper magnified and legible view. This is part 2 of Figure S9C].

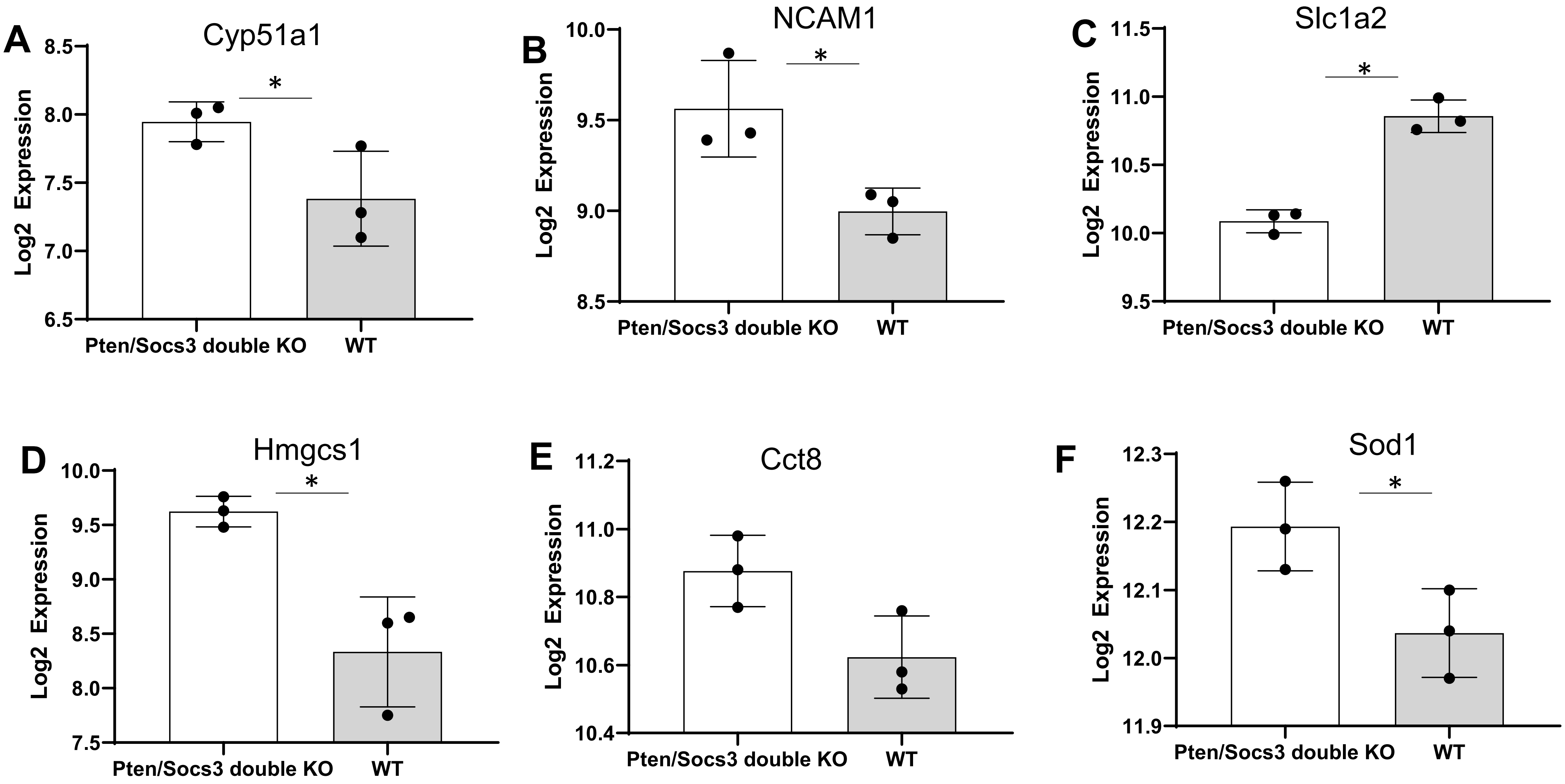

Figure S10, related to Figures 4 and 5. Expression of growth cone lipid-correlated genes in response to the Pten/Socs3(-/-) model of axon regeneration. (**A-F**) Expression of select early and late development lipid-correlated proteins in response to double knockouts of Pten, a negative regulator of mammalian target of rapamycin (mTOR), and Socs3, a negative regulator of Janus kinase/signal transducers and activators of transcription (JAK/STAT) pathway, in adult mice retinal ganglion cells (GSE32309). Stars indicate significant differences (**p*<0.05, compared to wildtype).

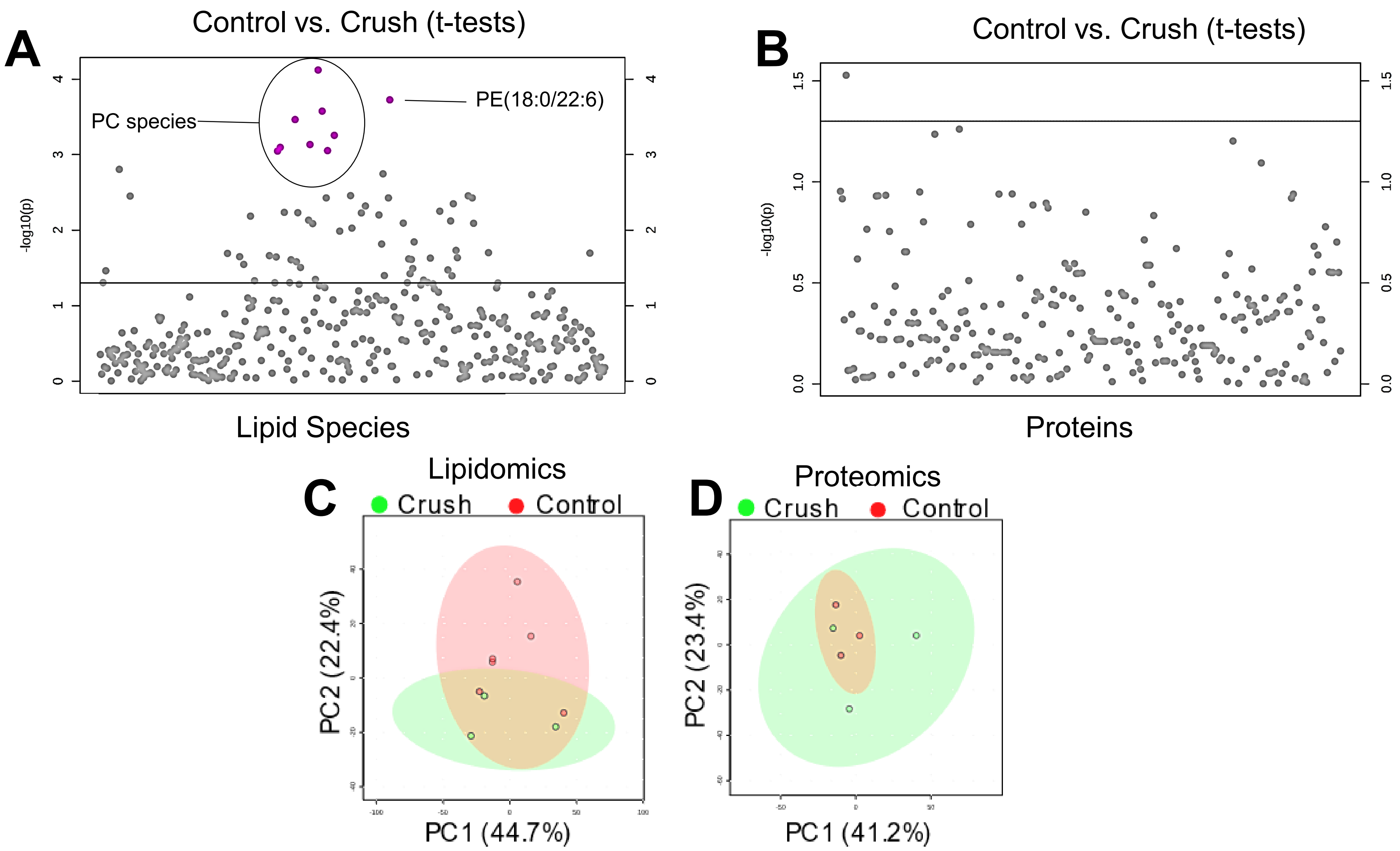

Figure S11, related to Figure 5. Lipidomic and transcriptomic analysis of crush vs control in mouse optic nerve. (A-B) T-tests with FDR adjusted p-values of lipid species (**A**) and proteins (PRIDE accession number PXD016197) (**B**) before and after optic nerve crush. FDR adjusted p-values threshold was set at 0.05. Pink values indicate significantly different. PC, phosphatidylcholine; PE, phosphatidylethanolamine. (C-D) Principle component analysis of crush vs. control lipidomic (**C**) and proteomic (**D**) data.

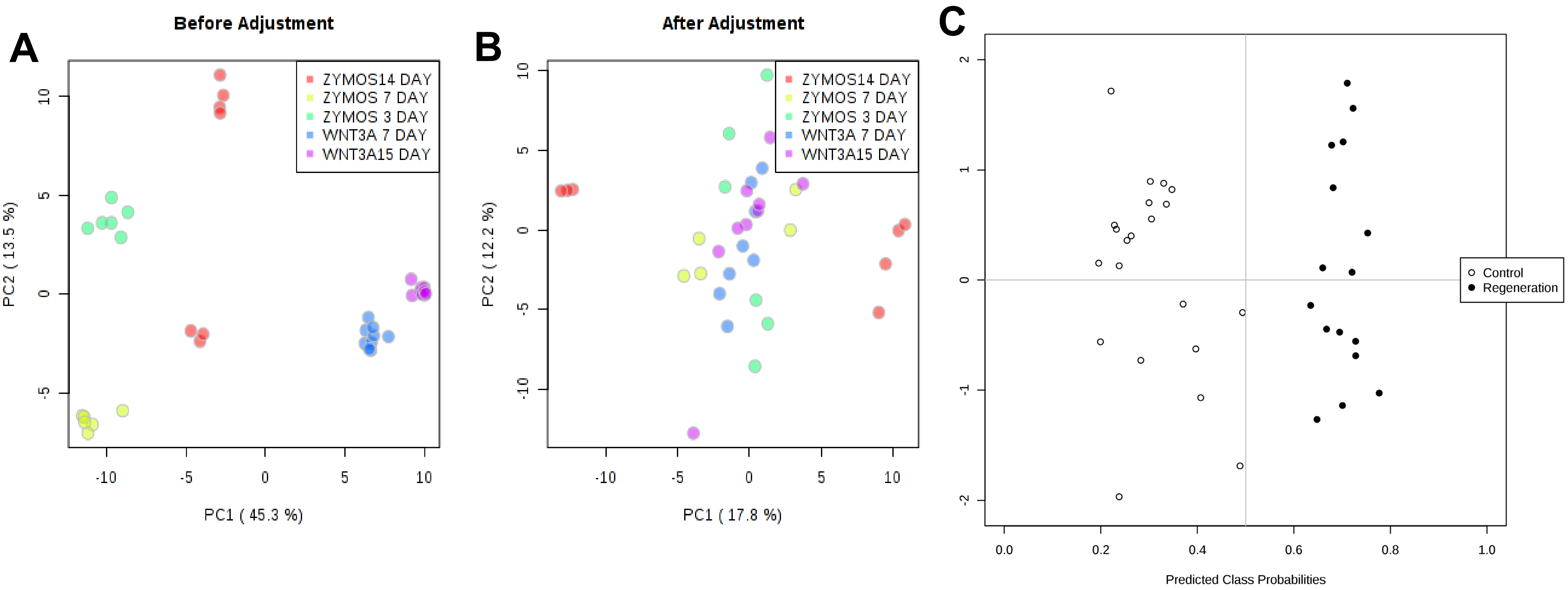

Figure S12, related to Figure 5. Batch correction and predicted class probabilities based on AUC values. (A-B) Principle component analysis of all lipidomic optic nerve regeneration datasets before (**A**) and after (**B**) batch correction adjustment using ComBat method.[38] (**C**) Predicted class probabilities for optic nerve lipidomic mouse samples (control and regeneration) constructed from a single marker: Cer(d18:1/24:0). This model placed 100% of the samples in the correct group.

Table S1. List of samples, related to Figure 1.

Fraction	Age	Molecules Analyzed	Biological replicate
Growth cone membrane (GCM)	E18	Protein/lipids	1–5/1-6
	P0	Protein/lipids	1–6/1-6
	P3	Protein/lipids	1–6/1-6
	P6	Protein/lipids	1–6/1-6
	P9	Protein/lipids	1–6/1-6
Growth cone particulate (GCP)	E18	Protein/lipids	1–6/1-6
	P0	Protein/lipids	1–6/1-6
	P3	Protein/lipids	1–6/1-6
	P6	Protein/lipids	1–6/1-6
	P9	Protein/lipids	1–5/1-6
